# CCR2 defines *in vivo* development and homing of IL-23-driven GM-CSF-producing Th17 cells

**DOI:** 10.1038/ncomms9644

**Published:** 2015-10-29

**Authors:** Ervin E. Kara, Duncan R. McKenzie, Cameron R. Bastow, Carly E. Gregor, Kevin A. Fenix, Abiodun D. Ogunniyi, James C. Paton, Matthias Mack, Diana R. Pombal, Cyrill Seillet, Bénédicte Dubois, Adrian Liston, Kelli P. A. MacDonald, Gabrielle T. Belz, Mark J. Smyth, Geoffrey R. Hill, Iain Comerford, Shaun R. McColl

**Affiliations:** 1 Department of Molecular and Cellular Biology, School of Biological Sciences, University of Adelaide, Adelaide, South Australia 5005, Australia; 2 Research Centre for Infectious Diseases, School of Biological Sciences, University of Adelaide, Adelaide, South Australia 5005, Australia; 3 Department of Internal Medicine II, University Hospital Regensburg, Regensburg 93042, Germany; 4 Department of Microbiology and Immunology, VIB and University of Leuven, B-3000 Leuven, Belgium; 5 Division of Molecular Immunology, Walter and Eliza Hall Institute of Medical Research, Parkville, Victoria 3052, Australia; 6 Department of Neurosciences, KU-Leuven–University of Leuven, B-3000 Leuven, Belgium; 7 QIMR Berghofer Medical Research Institute, Herston, Queensland 4006, Australia; 8 Department of Medical Biology, University of Melbourne, Parkville, Victoria 3010, Australia; 9 School of Medicine, University of Queensland, Herston, Queensland 4006, Australia; 10 The Royal Brisbane and Women's Hospital, Herston, Queensland 4029, Australia; 11 Centre for Molecular Pathology, School of Biological Sciences, University of Adelaide, Adelaide, South Australia 5005, Australia

## Abstract

IL-17-producing helper T (Th17) cells are critical for host defense against extracellular pathogens but also drive numerous autoimmune diseases. Th17 cells that differ in their inflammatory potential have been described including IL-10-producing Th17 cells that are weak inducers of inflammation and highly inflammatory, IL-23-driven, GM-CSF/IFNγ-producing Th17 cells. However, their distinct developmental requirements, functions and trafficking mechanisms *in vivo* remain poorly understood. Here we identify a temporally regulated IL-23-dependent switch from CCR6 to CCR2 usage by developing Th17 cells that is critical for pathogenic Th17 cell-driven inflammation in experimental autoimmune encephalomyelitis (EAE). This switch defines a unique *in vivo* cell surface signature (CCR6^−^CCR2^+^) of GM-CSF/IFNγ-producing Th17 cells in EAE and experimental persistent extracellular bacterial infection, and in humans. Using this signature, we identify an IL-23/IL-1/IFNγ/TNFα/T-bet/Eomesodermin-driven circuit driving GM-CSF/IFNγ-producing Th17 cell formation *in vivo*. Thus, our data identify a unique cell surface signature, trafficking mechanism and T-cell intrinsic regulators of GM-CSF/IFNγ-producing Th17 cells.

## Abstract (web summary)

Little is known regarding migration of Th17 cells that produce distinct cytokines implicated in protection and pathology. Kara *et al.* show that a switch from CCR6 to CCR2 by Th17 cells defines a signature (CCR6^−^CCR2^+^) of GM-CSF^+^ Th17 cells and drives pathology in a mouse model of autoimmunity.

## Graphical abstract

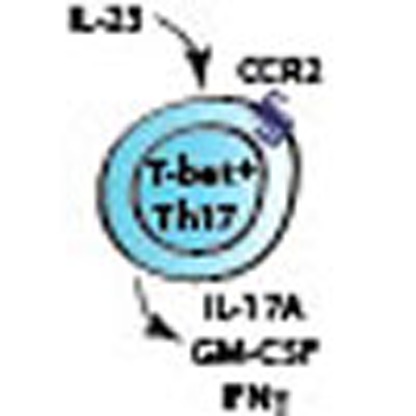

An emerging concept in inflammatory T-cell biology is the existence of a spectrum of T-helper 17 (Th17) phenotypes that vary in inflammatory potential. In autoimmunity, Th17 cell subsets that differ both in their developmental requirements and function have been described^1^. Transforming growth factor-β1 (TGFβ1) and interleukin-6 (IL-6) drive differentiation of IL-10-producing Th17 cells^2^,^3^,^4^, which are weak inducers of inflammation^2^,^3^,^4^ and can possess regulatory function^2^,^5^. Conversely, differentiation and effector function of Th17 cells with pathogenic function is dependent on IL-23 (refs 3, 4, 6, 7, 8, 9, 10), which induces expression of the effector cytokines granulocyte–macrophage-stimulating factor (GM-CSF) and interferon-γ (IFNγ)^6^,^7^,^11^. It is widely appreciated that IL-23-dependent Th17 cell responses orchestrate numerous CD4^+^ T-cell-driven pathologies including experimental autoimmune encephalomyelitis (EAE), the mouse model of multiple sclerosis (MS)^6^,^7^,^12^. It has been hypothesized that these two arms of the Th17 cell response evolved to coordinate different domains of protective immunity^12^ wherein Th17 cells with a more limited inflammatory potential mediate maintenance of barrier tissue integrity^2^,^5^,^13^, whereas more inflammatory subsets of Th17 cells amplify inflammation during persistent extracellular bacterial/fungal infection^14^,^15^. Although these models of Th17 cell biology are a useful construct for conceptualizing how different Th17 cell phenotypes participate in protective/pathological immune responses, present knowledge of distinct Th17 cell phenotypes has predominantly flowed from *in vitro*-based systems and is therefore limited. Understanding mechanisms governing development and trafficking of Th17 cells with pathogenic function during autoimmune inflammation is of critical importance as intervention of these processes presents as a tractable target for novel therapeutics.

Migratory properties of effector Th cells are imprinted during differentiation with induction of chemokine receptors that enable their differential trafficking to inflammatory lesions. CCR6 is a homing receptor shared by Th17 and regulatory T cells (Tregs)^16^, hypothesized to ensure that Th17 cell responses are closely regulated by Tregs to limit superfluous, and potentially damaging, inflammation^17^. However, emerging evidence suggests the existence of additional, more critical receptors in Th17 migration. In EAE, a ‘two-wave' model for encephalitogenic Th17-cell recruitment to the central nervous system (CNS) has been proposed where, in the first wave, CCR6 facilitates entry into the uninflamed CNS, followed by subsequent waves of CCR6-independent Th17 cell trafficking into the inflamed CNS^18^. Conversely, more recent studies have demonstrated that CCR6 is largely dispensable for EAE pathogenesis^19^,^20^, suggesting that recruitment of encephalitogenic Th17 cells to the CNS is CCR6 independent. However, the molecular basis for CCR6-independent trafficking of Th17 cells is unknown and migratory receptors that differentially recruit Th17 and Tregs to inflammatory lesions have not been identified.

Here we demonstrate that CCR2, not CCR6, is a key driver of encephalitogenic Th17 cell recruitment into the CNS. Further, we identify GM-CSF/IFNγ-producing Th17 cells in EAE and persistent extracellular bacterial infection as bearing a CCR6^−^CCR2^+^ phenotype in mice and in humans. Conversely, Th17 cells with an IL-10^+^ and IL-9^+^ cytokine profile, consistent with published descriptions of Th17 cells of more limited pathogenic potential, bear a CCR6^+^CCR2^+^ phenotype *in vivo*. Using these signatures, we demonstrate that an IL-23/IL-1/IFNγ/tumour necrosis factor-α (TNFα)/T-bet/Eomesodermin-driven circuit drives GM-CSF/IFNγ-producing Th17 cell development *in vivo*. Thus, we report a unique cell surface signature and novel developmental features of GM-CSF/IFNγ-producing Th17 cells *in vivo* and resolve the outstanding question regarding the molecular control of encephalitogenic Th17 cell trafficking to the CNS in EAE.

## Results

### Th17 cells express functional CCR2 during inflammation

**Figure 1:**
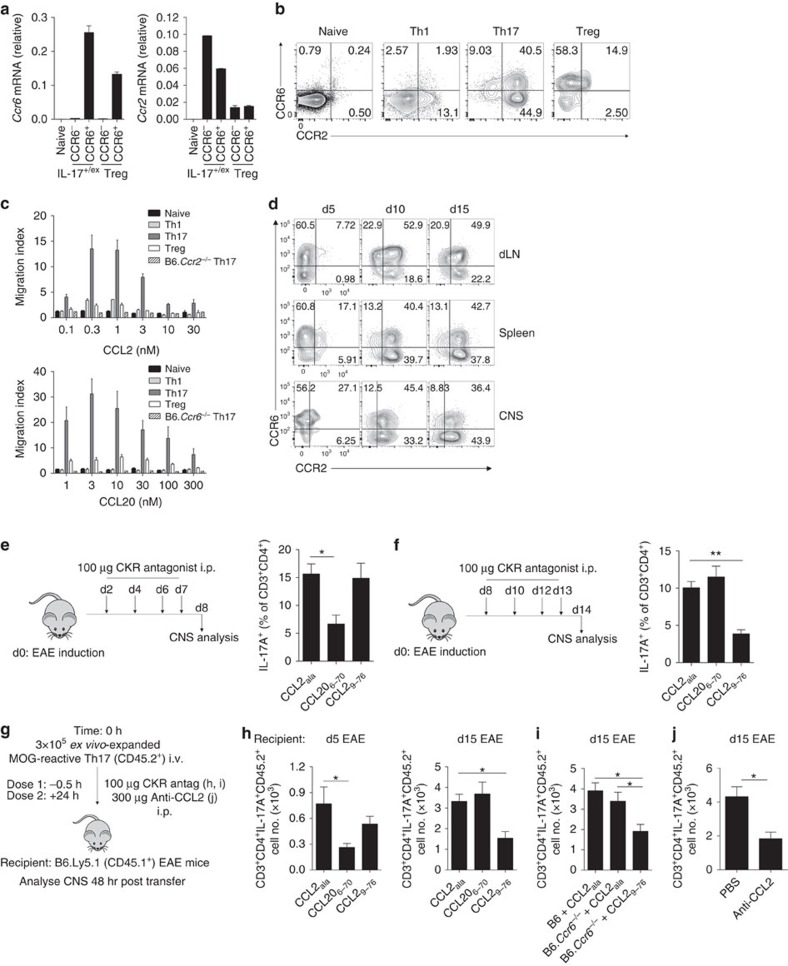
Th17 cell recruitment to the CNS is temporally regulated by CCR6 and CCR2. (**a**) Quantitative PCR of *Ccr6* and *Ccr2* transcript in CCR6^+^ and CCR6^−^ subsets of CD4^+^IL-17A^+/ex^ (currently, or previously Th17) cells (CD3^+^CD4^+^CD44^hi^IL-17AeYFP^+^–B6.*Il17a*^Cre^*Rosa26*^eYFP^ mice) and Tregs (CD3^+^CD4^+^Foxp3GFP^+^–B6.*Foxp3*^GFP^ mice) from the spleen/draining lymph node (dLN) of 5–6 mice d10 post MOG/CFA immunization. Data presented relative to *Rplp0* (mean±s.d.). (**b**) Representative flow cytometric analysis of CCR6/CCR2 staining on naive CD4^+^ (CD3^+^CD4^+^CD44^lo^), Th1 (CD3^+^CD4^+^CD44^hi^IL-17A^−^IFNγ^+^), Th17 (CD3^+^CD4^+^CD44^hi^IL-17A^+^) and Tregs (CD3^+^CD4^+^Foxp3^+^) from the spleen of B6 mice d10 post MOG/CFA immunization. Data are representative of three independent experiments with *n*=3–4 mice per experiment. (**c**) Transwell chemotaxis to CCL20 and CCL2 by indicated T-cell subsets from d10 MOG/CFA-immunized B6 mice. Th17 cells from B6.*Ccr6*^−/−^ and B6.*Ccr2*^−/−^ mice served as CCL20 and CCL2 controls, respectively. Data are representative of two independent experiments with *n*=4 mice per experiment. (**d**) Representative flow cytometric analysis of CCR6/CCR2 staining on Th17 cells (CD3^+^CD4^+^CD44^hi^IL-17A^+^) in the dLN, spleen and CNS on d5, 10 and 15 post EAE induction. Data are representative of four independent experiments with *n*=4–6 mice per timepoint. (**e**,**f**) EAE-immunized B6 mice were administered 100 μg of CCL2_ala_ (scrambled peptide control; *n*=5), CCL20_6–70_ (CCR6 antagonist; *n*=4) or CCL2_9–76_ (CCR2 antagonist; *n*=5) i.p. on days 2, 4, 6 and 7 (**e**) or days 8, 10, 12 and 13 (**f**). CNS-infiltrating Th17 cells were quantified 24 h after the final antagonist treatment. (**g**) Schematic of Th17 cell transfer system. (**h**) Number of transferred Th17 cells (CD3^+^CD4^+^IL-17A^+^CD45.2^+^) in CNS 48 h post transfer from CCL20_6–70_ (*n*=5), CCL2_9–76_ (*n*=5) or CCL2_ala_ (*n*=5) treated B6.Ly5.1 recipients pre-immunized for EAE 5 (left) or 15 (right) days prior. (**i**) Number of transferred CD45.2^+^ B6 (*n*=5) or B6.*Ccr6*^−/−^ Th17 cells in CNS 48 h post transfer of CCL2_ala_ (*n*=6)- or CCL2_9–76_ (*n*=6)-treated B6.Ly5.1 recipients pre-immunized for EAE 15 days prior. (**j**) Number of transferred CD45.2^+^ Th17 cells in CNS 48 h post transfer of PBS (*n*=5)- or anti-CCL2 (*n*=5)-treated B6.Ly5.1 recipients pre-immunized for EAE 15 days prior. (**c**,**e**,**f**,**h**,**i**,**j**) Data are presented as mean±s.e.m. (**e**,**f**,**h**–**j**) *P≤0.05, **P≤0.01; (**e**,**f**,**h**) one-way analysis of variance (ANOVA) with Dunnett's multiple comparisons test relative to control CCL2_ala_-treated group; (**i**) one-way ANOVA with Bonferroni multiple comparisons test; (**j**) unpaired two-tailed Student's *t*-test.

To identify CCR6-independent mechanisms mediating recruitment of Th17 cells and to compare migratory potential of Th17 and Tregs, we screened for the expression of all known chemokine receptors in CCR6^+^ and CCR6^−^ subsets of Tregs from B6.*Foxp3*^GFP^ mice and IL-17A-eYFP^+^CD4^+^ T cells from B6.*Il17a*^Cre^*Rosa26*^eYFP^ mice, in which Cre recombinase is driven by *Il17a* promoter activity to permanently mark cells that are currently producing or have previously expressed IL-17A (IL-17A^+/ex^) with enhanced yellow fluorescent protein (eYFP)^11^ (Supplementary Fig. 1). Notably, high levels of *Ccr2* messenger RNA were apparent in CCR6^−^CD4^+^IL-17A^+/ex^ cells (Fig. 1a). CCR2 protein was minimally expressed by naive, Th1 and Treg populations from EAE-induced wild-type (WT) mice, whereas IL-17A-producing CD4^+^ T cells, hereafter termed Th17 cells, expressed either CCR6 and/or CCR2 (CCR6^+^CCR2^−^, CCR6^+^CCR2^+^ or CCR6^−^CCR2^+^) (Fig. 1b). Functionally, *ex vivo* transmigration assays demonstrated that Th17 cells were the most CCL2-responsive CD4^+^ T-cell subset from EAE mice (Fig. 1c). In the CNS during EAE, the first detectable Th17 cells (day (d)5 post immunization) were predominantly CCR6^+^CCR2^−^; however, as disease progressed, CCR2-expressing Th17 cells bearing CCR6^+^CCR2^+^ or CCR6^−^CCR2^+^ phenotypes substantially increased in frequency (Fig. 1d). This was mirrored in secondary lymphoid organs (SLOs), as Th17 cells on d5 in the lymph node and spleen were predominantly CCR6^+^CCR2^−^, followed by the emergence of CCR6^+^CCR2^+^ and CCR6^−^CCR2^+^ Th17 cells by d10 post immunization (Fig. 1d). Thus, among the major CD4^+^ T-cell subsets in EAE, functional CCR2 expression is restricted to Th17 cells that arise following emergence of CCR6^+^ Th17 cells.

### CCR2 drives Th17 recruitment to the inflamed CNS

To map the role of CCR6 and CCR2 in temporal regulation of Th17 cell recruitment to the CNS during EAE, we treated mice with peptide antagonists for CCR6 (CCL20_6–70_)^21^,^22^ or CCR2 (CCL2_9–76_)^23^ during the pre-clinical or effector phases of disease. CCR6 antagonism reduced CNS accumulation of Th17 cells when administered during the pre-clinical phase, but did not alter Th17 cell population of the CNS when administered during the effector phase of disease (Fig. 1e,f). Conversely, CCR2 antagonism administered during the effector phase, but not the pre-clinical phase of disease, reduced Th17 cell population of the CNS (Fig. 1e,f). To extend these observations, we transferred *ex vivo*-expanded myelin oligodendrocyte glycoprotein (MOG)-reactive Th17 cells into B6.Ly5.1 recipients pre-immunized for EAE either 5 (pre-clinical) or 15 (chronic) days prior and concomitantly antagonized CCR6 or CCR2 (Fig. 1g). CCR6 antagonism inhibited CNS accumulation of transferred Th17 cells during the pre-clinical but not the chronic phase of EAE, whereas CCR2 antagonism only reduced transferred Th17 cell population of the CNS when administered during the chronic phase of disease (Fig. 1h). Furthermore, transferred *Ccr6*-deficient Th17 cells accumulated normally in the CNS of d15 pre-immunized recipients, but this was inhibited by concomitant antagonism of CCR2 (Fig. 1i). It has been reported that CCL2 levels in the CNS increase as EAE pathology transitions from pre-clinical to peak disease^24^, and CCL2 plays an important role in Th17 accumulation in the inflamed CNS, as transferred Th17 cells were less abundant in the CNS of CCL2-neutralized recipients (Fig. 1j). Collectively, these data indicate that CCR6 promotes recruitment of Th17 cells into the CNS at early phases of EAE, whereas CCR2/CCL2 drives Th17 cells into the CNS at later time points, during a CCR6-independent phase of their trafficking.

**Figure 2:**
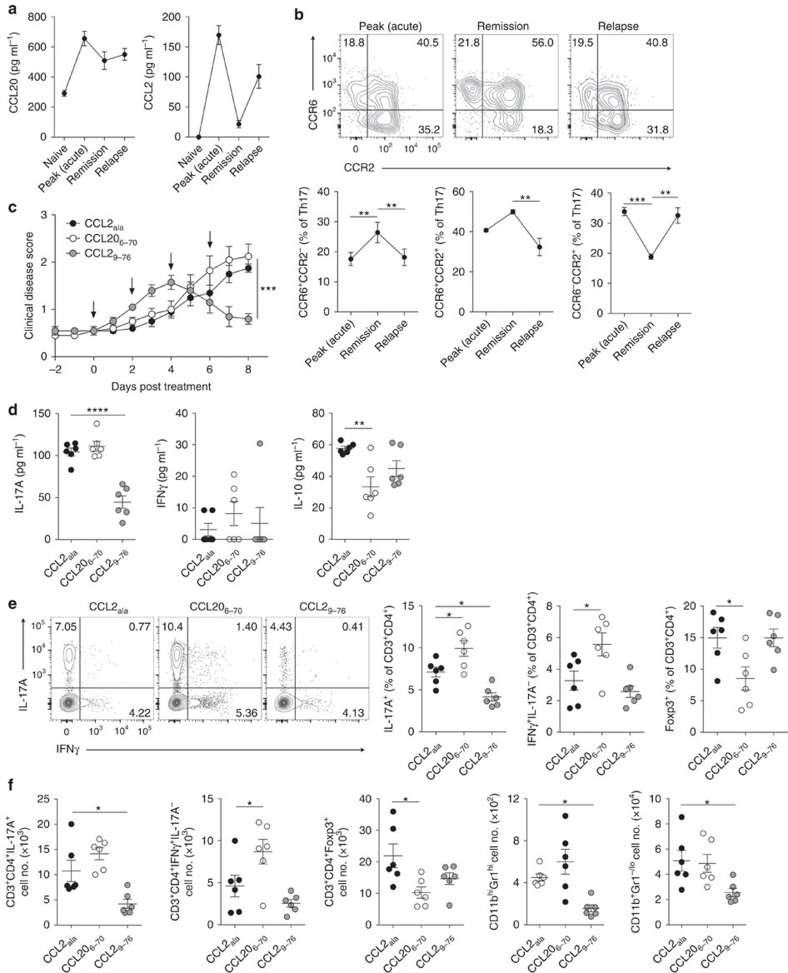
CCR2 promotes Th17 cell responses in EAE relapse. (**a**) CCL20 and CCL2 protein abundance in the CNS at indicated stages of EAE in SJL/J mice as determined by ELISA (*n*=5 mice per group). (**b**) Representative flow cytometric analysis and quantification of CCR6/CCR2 staining on CNS-infiltrating Th17 cells (CD3^+^CD4^+^IL-17A^+^) at peak acute disease (left), remission (middle) and relapse (right) of EAE-induced SJL/J mice. Data are representative of two independent experiments with *n*=6 per experiment. (**c**) Clinical disease scores of EAE relapse in SJL/J mice treated with CCL20_6–70_ (*n*=10), CCL2_9–76_ (*n*=10) or CCL2_ala_ (*n*=10) i.p. on days indicated by black arrows. Treatment began on the fourth day of remission (disease score ≤1 after reaching ≥2 prior; d0 on graph). (**d**) IL-17A, IFNγ and IL-10 protein abundance in the CNS of mice on d8 following treatment in **c** as determined by ELISA (*n*=6 per group). (**e**) Representative flow cytometric analysis of IL-17A and IFNγ staining on CNS-infiltrating CD3^+^CD4^+^ cells on d8 following treatment in **c** (*n*=6 per group). Right, quantification of IL-17A^+^ (Th17), IL-17A^−^IFNγ^+^ (Th1) and Foxp3^+^ (Tregs) cells among CNS-infiltrating CD3^+^CD4^+^ cells. (**f**) Total number of CNS-infiltrating Th17, Th1, Treg cells, Gr1^+^ leukocytes and other myeloid cells (CD11b^+^Gr1^lo/−^) on d8 following treatment in **c** (*n*=6 per group). (**a**–**f**) Data are presented as mean±s.e.m.; *P≤0.05, **P≤0.01, ***P≤0.001. (**d**–**f**) Each dot represents an individual mouse. (**b**) One-way analysis of variance (ANOVA) with Bonferroni multiple comparisons test. (**c**) Two-way ANOVA with multiple comparisons test. (**d**–**f**) One-way ANOVA with Dunnett's multiple comparisons test relative to control CCL2_ala_-treated group.

Having identified CCR2, and not CCR6, as a key receptor driving Th17 cell recruitment to the inflamed CNS in chronic EAE, we next assessed CCR6 and CCR2 function in a model of relapsing–remitting EAE. CCL20 was detectable in the CNS at homeostasis, increased during acute disease and remained abundant during remission and relapse (Fig. 2a). Conversely, CCL2 was undetectable in the uninflamed CNS and low during remission, and was most abundant during acute disease and in EAE relapse (Fig. 2a). In keeping with CNS chemokine expression, frequencies of CCR6^−^CCR2^+^ Th17 cells were highest during peak acute disease and relapse, whereas CCR6-expressing populations of Th17 cells were more abundant during remission (Fig. 2b). To assess the function of CCR6 and CCR2 in relapse, we treated mice during EAE remission with CCR6 or CCR2 peptide antagonists and assessed molecular, cellular and clinical manifestations of disease relapse. Notably, CCR6 antagonism did not alter the incidence or severity of EAE relapse (Fig. 2c) and led to reduced CNS levels of IL-10, fewer CNS-infiltrating Tregs and augmented CNS-infiltrating Th17 cells and Gr1^+^ leukocytes (mostly neutrophils based on scatter analysis) (Fig. 2d–f). In contrast, CCR2 antagonism dampened EAE relapse severity (Fig. 2c) with less IL-17A in the CNS and reduced CNS-infiltrating Th17 cells (Fig. 2d–f). Fewer CNS-infiltrating Gr1^+^ leukocytes and other CD11b^+^ myeloid cells were also detected in CCR2 antagonized mice (Fig. 2f). Thus, CCR2 drives EAE relapse and promotes Th17 cell responses in the CNS, whereas CCR6 supports optimal Treg responses in these settings.

### CCR2 drives GM-CSF-producing Th17 cell homing to the CNS

**Figure 3:**
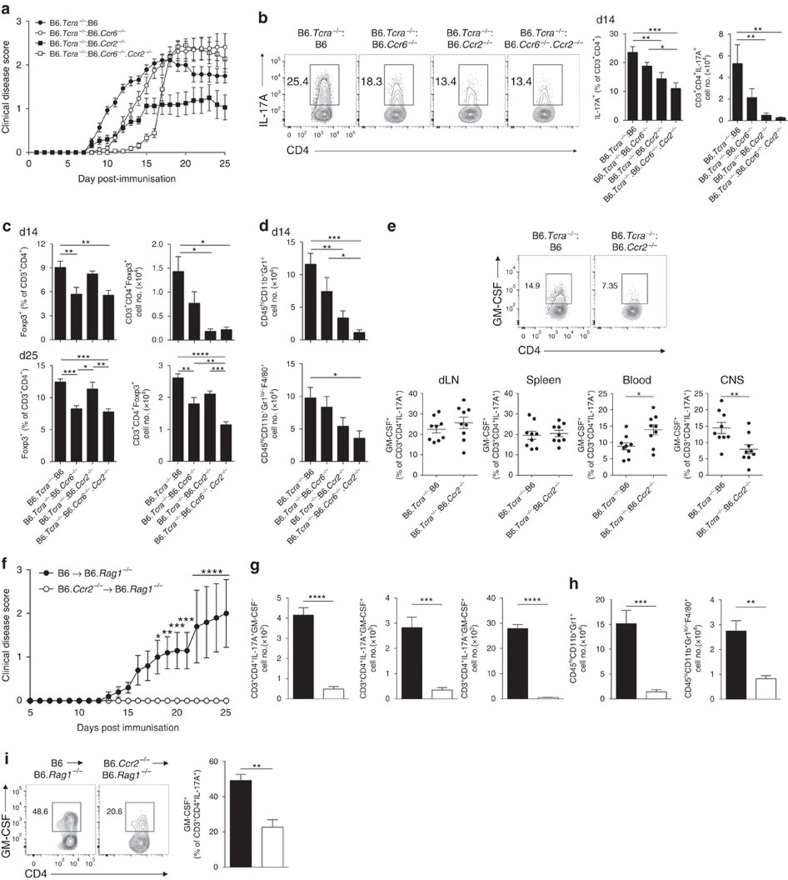
CCR2 drives recruitment of Th17 cells with pathogenic function into the inflamed CNS. (**a**) EAE clinical disease scores of T-cell-specific chemokine receptor-deficient bone marrow (BM) chimeras. T-cell-specific knockout (KO) chimeric mice were generated by transferring BM derived from B6.*Tcra*^−/−^ (80%) and B6 (*n*=16), B6.*Ccr6*^−/−^ (*n*=18), B6.*Ccr2*^−/−^ (*n*=16) or B6.*Ccr6*^−/−^.*Ccr2*^−/−^ (*n*=18) (20%) into lethally irradiated B6.Ly5.1 recipients. Data are pooled from two independent experiments. (**b**) Representative flow cytometric analysis and quantification of CNS-infiltrating Th17 cell frequencies in T-cell-specific KO chimeras on d14 EAE. B6 (*n*=9), B6.*Ccr6*^−/−^ (*n*=10), B6.*Ccr2*^−/−^ (*n*=9) and B6.*Ccr6*^−/−^.*Ccr2*^−/−^ (*n*=9). (**c**) Frequency and total number of CNS-infiltrating Tregs in T-cell-specific KO chimeras on d14 (top) and d25 (bottom) EAE. B6 (d14: *n*=9; d25: *n*=7), B6.*Ccr6*^−/−^ (d14: *n*=10; d25: *n*=8), B6.*Ccr2*^−/−^ (d14: *n*=9; d25: *n*=7) and B6.*Ccr6*^−/−^.*Ccr2*^−/−^ (d14: *n*=9; d25: *n*=9). (**d**) Number of Gr1^+^ leukocytes (top) and Gr1^lo^/-F4/80^+^ leukocytes (bottom) in the CNS of T-cell-specific KO chimeras on d14 EAE. B6 (*n*=9), B6.*Ccr6*^−/−^ (*n*=10), B6.*Ccr2*^−/−^ (*n*=9) and B6.*Ccr6*^−/−^.*Ccr2*^−/−^ (*n*=9). (**e**) Representative flow cytometric analysis and quantification of GM-CSF-producing Th17 cells (CD3^+^CD4^+^IL-17A^+^) in the draining lymph node (dLN), spleen, blood and CNS of B6 (*n*=9) and B6.*Ccr2*^−/−^ (*n*=9) T-cell chimeras 14 days post EAE induction. (**f**) EAE clinical disease score of B6.*Rag1*^−/−^ reconstituted with 8 × 10^6^ purified CD4^+^ T cells from B6 (*n*=5) or B6.*Ccr2*^−/−^ mice (*n*=5). Number of CNS-infiltrating (**g**) IL-17A^+^GM-CSF^−^, IL-17A^+^GM-CSF^+^ and IL-17A^−^GM-CSF^+^ CD4^+^ T cells, and (**h**) Gr1^+^ and Gr1^lo/−^F4/80^+^ leukocytes on d25 EAE in B6.*Rag1*^−/−^ reconstituted mice (black bars, B6; white bars, B6.*Ccr2*^−/−^). (**i**) Representative flow cytometric analysis and quantitation of GM-CSF-producing cells among CNS-infiltrated Th17 cells on d25 EAE of B6.*Rag1*^−/−^ reconstituted mice (black bars, B6; white bars, B6.*Ccr2*^−/−^). (**a**–**i**) Data are presented as mean±s.e.m.; *P≤0.05, **P≤0.01, ***P≤0.001, ****P≤0.0001. (**b**–**d**) One-way analysis of variance (ANOVA) with Bonferroni multiple comparisons test. (**e**,**g**–**i**) Unpaired two-tailed Student's *t*-test. (**f**) Two-way ANOVA with multiple comparisons test.

CCR2 has been previously shown to drive EAE pathogenesis^24^,^25^,^26^; however, a T-cell intrinsic role for CCR2 has not been clearly demonstrated. Thus, to specifically examine T-cell intrinsic functions of CCR6 and CCR2 in T-cell trafficking during EAE, we constructed bone marrow (BM) chimeras, reconstituting lethally irradiated B6.Ly5.1 recipients with 80% BM from B6.*Tcra*^−/−^ donors and 20% BM from either B6, B6.*Ccr6*^−/−^, B6.*Ccr2*^−/−^ or B6.*Ccr6*^−/−^.*Ccr2*^−/−^ donors. Notably, T-cell-specific deletion of *Ccr2* reduced CNS-infiltrating Th17 cells and diminished EAE severity (Table 1 and Fig. 3a,b). In contrast, deletion of *Ccr6* delayed, but ultimately exacerbated EAE without substantially altering CNS-infiltrating Th17 cells, but reduced CNS-infiltrating Tregs at peak (d14) and chronic (d25) disease (Table 1 and Fig. 3a–c). Deletion of both *Ccr6* and *Ccr2* in T cells substantially delayed disease onset (Fig. 3a). However, akin to *Ccr6*-deficient T-cell chimeras, *Ccr6*^−/−^*Ccr2*^−/−^ T-cell chimeric mice ultimately manifest EAE, associated with fewer CNS-infiltrating Th17 cells at peak disease, but also reduced frequencies of CNS-infiltrating Treg cells at all time points assessed (Table 1 and Fig. 3a–c). Fewer CNS-infiltrating Gr1^+^ leukocytes were present in *Ccr2*-deficient and *Ccr6*/*Ccr2*-deficient T-cell chimeras at peak disease, consistent with diminished CNS Th17 cell responses in these mice (Fig. 3d). These data indicate that CCR2 plays a key role in mediating trafficking of T cells with pathogenic function to the CNS during EAE, whereas CCR6 functions as an important axis for Treg function in this model. Recent data have shown that encephalitogenic Th17 cells in EAE produce the inflammatory cytokine GM-CSF^6^,^7^. In keeping with this, T-cell-specific deletion of *Ccr2* reduced GM-CSF^+^ Th17 cell abundance in the CNS without altering their development in SLOs (Fig. 3e). Further, GM-CSF-producing Th17 cells were more abundant in circulation, suggesting that CCR2 drives circulation-to-CNS trafficking of encephalitogenic Th17 cells (Fig. 3e). To more definitively address this point, we transferred purified *Ccr2*-deficient CD4^+^ T-cells into B6.*Rag1*^−/−^ recipients and induced EAE. In this model, Th17 cells with pathogenic function that arise from transferred CD4^+^ T cells represent the critical disease-initiating cell type^7^. Strikingly, recipient mice receiving *Ccr2*-deficient T cells were resistant to EAE (Table 2 and Fig. 3f), exemplifying the critical requirement for CCR2 in encephalitogenic T-cell function in this model. Furthermore, although Th17 cell frequencies were equivalent in SLOs (Supplementary Fig. 2), Th17 cells and GM-CSF^+^ CD4^+^ T cells were markedly reduced in the CNS of B6.*Rag1*^−/−^ recipients reconstituted with *Ccr2*-deficient CD4^+^ T cells (Fig. 3g). Accordingly, fewer CNS-infiltrating Gr1^+^ and Gr1^lo/−^F4/80^+^ leukocytes were present in these mice (Fig. 3h). Importantly, GM-CSF-producing Th17 cells were substantially reduced in the CNS in the absence of CCR2 (Fig. 3i). Collectively, these data indicate that CCR2 drives CNS accumulation of Th17 cells with pathogenic function.

### CCR6^−^CCR2^+^ defines GM-CSF/IFNγ-producing Th17 cells

**Figure 4:**
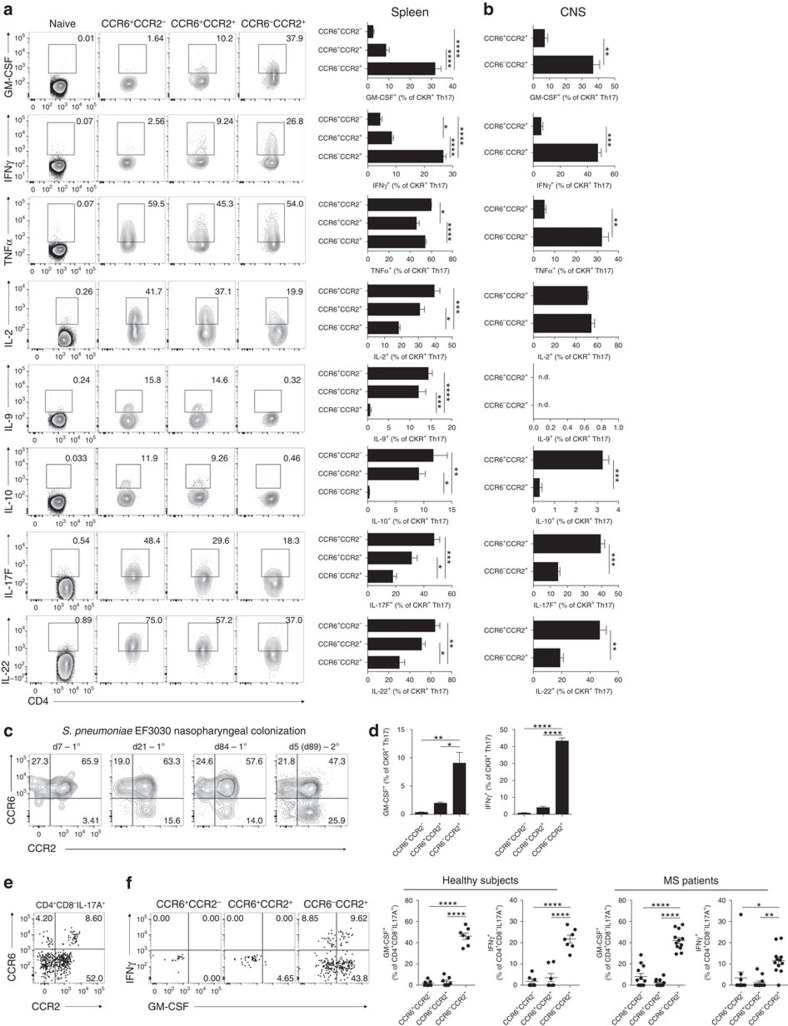
The CCR6^−^CCR2^+^ signature defines murine and human GM-CSF/IFNγ-producing Th17 cells *in vivo*. (**a**) Representative flow cytometric analysis and quantification of GM-CSF, IFNγ, TNFα, IL-2, IL-9, IL-10, IL-17F and IL-22 staining in naive CD4^+^ (CD3^+^CD4^+^CD44^lo^), CCR6^+^CCR2^−^, CCR6^+^CCR2^+^ and CCR6^−^CCR2^+^ Th17 cell populations (CD3^+^CD4^+^CD44^hi^IL-17A^+^) from the spleen of mice immunized for EAE 10 days prior. Data representative of three independent experiments with *n*=4–5 mice per experiment. (**b**) Percent cytokine positive among CCR6^+^CCR2^+^ and CCR6^−^CCR2^+^ Th17 cells (CD3^+^CD4^+^CD44^hi^IL-17A^+^) in the CNS d15 post EAE induction. Data are pooled from three independent experiments with *n*=8 CNS pooled per experiment. (**c**) Representative flow cytometric analysis of CCR6/CCR2 staining on Th17 cells (CD3^+^CD4^+^IL-17A^+^) in the spleen of B6 mice colonized in the nasopharynx with *S. pneumoniae* strain EF3030 7, 21 and 84 days post primary (1^o^) immunization and 5 days (d89) post secondary (2^o^) immunization. Data are representative of *n*=5–6 mice per timepoint. (**d**) Expression of GM-CSF and IFNγ among CCR6^+^CCR2^−^, CCR6^+^CCR2^+^ and CCR6^−^CCR2^+^ Th17 cell populations (CD3^+^CD4^+^IL-17A^+^) from the spleen of B6 mice 21 days post 1^o^ *S. pneumoniae* strain EF3030 nasopharyngeal colonization (*n*=4). (**e**) Representative flow cytometric analysis of CCR6/CCR2 staining on circulating human Th17 cells (CD4^+^CD8^−^IL-17A^+^) from an MS patient. (**f**) Representative flow cytometric analysis and quantification of IFNγ and GM-CSF staining on CCR6^+^CCR2^−^, CCR6^+^CCR2^+^ and CCR6^−^CCR2^+^ human Th17 cell subsets from the peripheral blood of healthy (*n*=7) and MS patients (*n*=12). (**a**,**b**,**d**,**f**) Data are presented as mean±s.e.m.; *P≤0.05; **P≤0.01; ***P≤0.001; ****P≤0.0001; one-way analysis of variance with Bonferroni multiple comparisons test.

Recent work has demonstrated that a shift to GM-CSF- and IFNγ-secreting capability enhances the pathogenicity of Th17 cells^3^,^6^,^7^,^11^,^27^. Having identified CCR2 as a key receptor driving GM-CSF-producing encephalitogenic Th17 cell trafficking in EAE, we next examined a possible relationship between the cytokine-secreting repertoire of Th17 cells and CCR6/CCR2-expressing Th17 cell types. Strikingly, expression of GM-CSF and IFNγ was most abundant in CCR6^−^CCR2^+^ Th17 cells (Fig. 4a), which also expressed the highest level of TNFα in the CNS (Fig. 4b). Conversely, IL-10 and IL-9 were confined to CCR6-expressing Th17 cells (Fig. 4a,b). IL-2 was most abundant in CCR6^+^ Th17 cells in the spleen, although expression in the CNS at peak disease was equally distributed between CCR6^+^CCR2^+^ and CCR6^−^CCR2^+^ populations (Fig. 4a,b). CCR6^−^CCR2^+^ Th17 cells expressed less IL-22 and IL-17F than CCR6^+^ populations (Fig. 4a,b). These data indicate that Th17 cell CCR6/CCR2 expression status can delineate distinct cytokine-secreting phenotypes of Th17 cells *in vivo*. Specifically, CCR6^−^CCR2^+^ defines GM-CSF/IFNγ-producing Th17 cells *in vivo* previously described to possess pathogenic function in EAE^3^,^6^,^7^,^27^, whereas CCR6^+^CCR2^+^ Th17 cells express a distinct cytokine-secreting repertoire, including IL-10 and IL-9, consistent with descriptions of Th17 cells with a more limited pathogenic potential^2^,^3^,^4^,^5^. CCR6^+^CCR2^−^ Th17 cells that predominate in the early stages of EAE express a diverse cytokine profile including both inflammatory (IL-17A/F, TNFα, IL-22 and IL-2) and regulatory (IL-10) cytokines.

To determine whether these observations also applied in infectious settings, we examined Th17 cells generated in a model of persistent *Streptococcus pneumoniae* nasopharyngeal colonization. Colonization using *S. pneumoniae* strain EF3030 induces long-term focal infection that resolves in B6 mice by 4 weeks post inoculation^28^. Importantly, protection against *S. pneumoniae* nasopharyngeal colonization has been shown to require Th17 cells^29^ and GM-CSF-producing T cells are also produced in response to this infection^30^. *S. pneumoniae*-induced Th17 cells were detectable in the spleen by day 7, peaked at d21, remained above baseline 84 days post primary infection and were substantially expanded 5 days post reinfection (Supplementary Fig. 3). The majority of initial (d7 post inoculation) Th17 cells generated in response to infection expressed CCR6 and were followed by the later emergence of CCR6^−^CCR2^+^ Th17 cells by d21 post infection (Fig. 4c). CCR6^−^CCR2^+^ Th17 cells were still detectable 84 days post primary infection and were substantially expanded 5 days post secondary infection (Fig. 4c), indicating that CCR6^−^CCR2^+^ Th17 cells contribute to the memory compartment in this model. Importantly, Th17 populations generated in response to persistent bacterial infection displayed similar cytokine-secreting repertoires as observed in EAE, as GM-CSF^+^ or IFNγ^+^ Th17 cells were found almost exclusively in the CCR6^−^CCR2^+^ population (Fig. 4d). Thus, CCR6^−^CCR2^+^ defines the GM-CSF/IFNγ-producing population of Th17 cells that arise in a model of persistent extracellular bacterial infection.

GM-CSF- or IFNγ-producing Th17 cells are enriched in active MS brain lesions^31^,^32^. Thus, we next examined whether a similar relationship between expression of these cytokines and CCR2/CCR6 cell surface status existed in human Th17 cells from healthy and MS patients. CCR6- and/or CCR2-positive populations of Th17 cells were detected in the peripheral blood of both healthy and MS patients, with the majority of these cells bearing a CCR6^−^CCR2^+^ phenotype (Fig. 4e). As in mice, human Th17 cell expression of GM-CSF and IFNγ was confined to CCR6^−^CCR2^+^ populations in both healthy subjects and MS patients (Fig. 4f). The presence of CCR6^−^CCR2^+^ Th17 cells in healthy subjects was not unexpected given the ability of this subset to enter memory in response to infection (Fig. 4c,d). Thus, the CCR6^−^CCR2^+^ signature also defines human GM-CSF/IFNγ-producing Th17 cells.

### Differentiation of CCR6^−^CCR2^+^ Th17 cells *in vivo*

**Figure 5:**
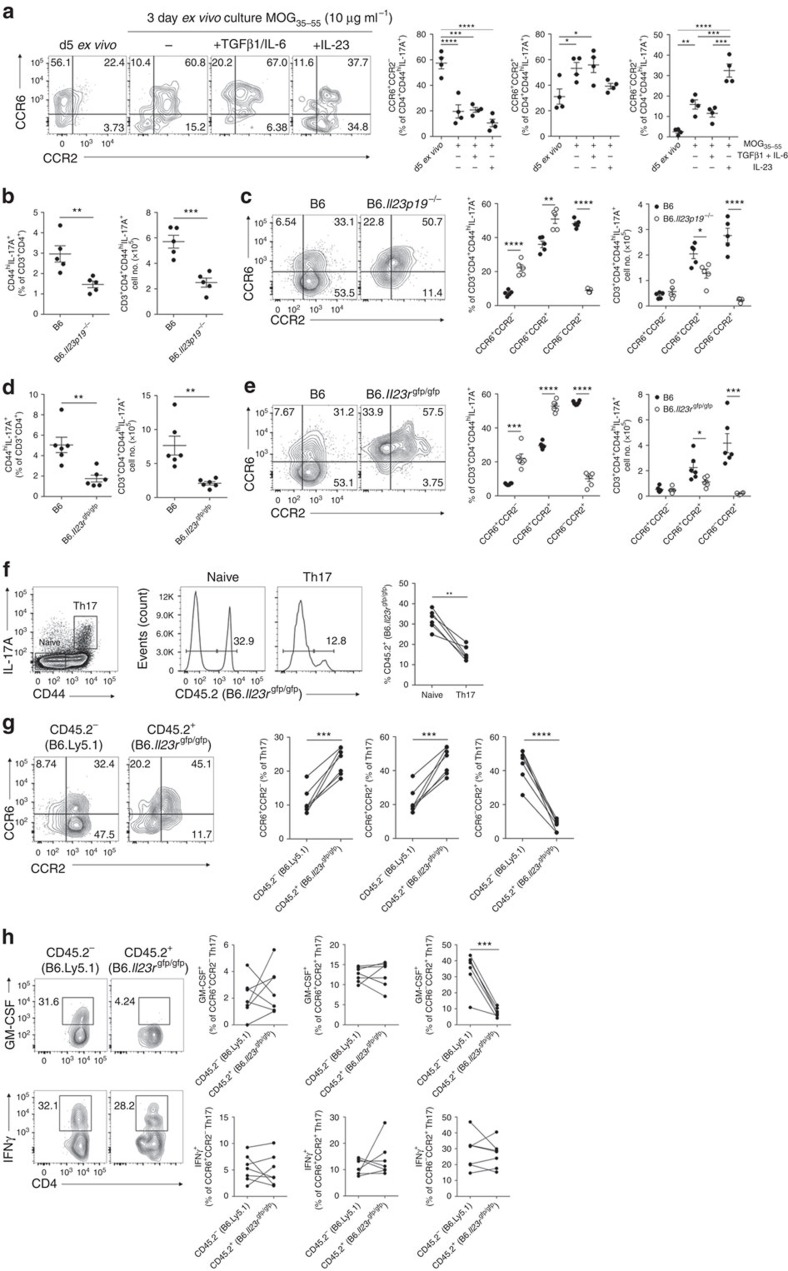
IL-23 drives differentiation of CCR6^−^CCR2^+^ Th17 cells *in vivo*. (**a**) Representative flow cytometric analysis and quantification of CCR6/CCR2 staining on Th17 cells (CD3^+^CD4^+^CD44^hi^IL-17A^+^) 5 days post MOG/CFA immunization (d5 *ex vivo*) and after 3 days *ex-vivo* culture with MOG_35–55_ in the presence of either no cytokines (−), TGFβ1/IL-6 or IL-23. Data are representative of two independent experiments, *n*=4. (**b**,**c**) Analysis of B6 (*n*=5) and B6.*Il23p19*^−/−^ (*n*=5) mice d10 post MOG/CFA immunization. (**b**) Frequency and total number of Th17 cells in the spleen; (**c**) representative flow cytometric analysis and quantification of CCR6/CCR2 staining on Th17 cells. (**d**,**e**) Analysis of B6 (*n*=6) and B6.*Il23r*^gfp/gfp^ (*n*=6) mice d10 post MOG/CFA immunization. (**d**) Frequency and total number of Th17 cells in the spleen; (**e**) representative flow cytometric analysis and quantification of CCR6 and CCR2 staining on Th17 cells. (**f**) Representative flow cytometric analysis and quantification of CD45.2^+^ (B6.*Il23r*^gfp/gfp^) cells (right) within naive CD4^+^ (CD3^+^CD4^+^CD44^lo^) and Th17 cells (CD3^+^CD4^+^CD44^hi^IL-17A^+^) (left) in the spleen of B6*.Il23r*^gfp/gfp^ mixed BM chimeric mice immunized with MOG/CFA 10 days prior (*n*=7). (**g**) Representative flow cytometric analysis and quantification of CCR6/CCR2 staining on CD45.2^−^ (B6.Ly5.1) and CD45.2^+^ (B6.*Il23r*^gfp/gfp^) Th17 cells in mixed BM chimeras immunized with MOG/CFA 10 days prior (*n*=7). (**h**) Representative flow cytometric analysis of GM-CSF and IFNγ staining among CD45.2^−^ (B6.Ly5.1) and CD45.2^+^ (B6.*Il23r*^gfp/gfp^) CCR6^−^CCR2^+^ Th17 cells in B6.*Il23r*^gfp/gfp^ (*n*=7) mixed BM chimeric mice 10 days post MOG/CFA immunization. Right, GM-CSF and IFNγ expression among CCR6/CCR2-expressing Th17 cell populations in B6.*Il23r*^gfp/gfp^ mixed BM chimeras. (**a**–**h**) Each dot represents an individual mouse; *P≤0.05; **P≤0.01, ***P≤0.001, ****P≤0.0001. (**a**–**d**) Data are presented as mean±s.e.m. (**a**) One-way analysis of variance with Bonferroni multiple comparisons test. (**b**–**e**) Unpaired two-tailed Student's *t*-test. (**f**–**h**) Paired two-tailed Student's *t*-test.

Our data suggested that the ‘switch' from CCR6 to CCR2 usage by developing Th17 cells was coupled with induction of a cytokine-secreting profile reported to promote Th17 cell pathogenicity in EAE^3^,^6^,^7^,^27^. Differentiation of Th17 cells from naive precursors and their subsequent acquisition of pathogenicity are coordinated by various distinct cytokine signals^12^. It has been reported that initial Th17 cell differentiation *in vivo* occurs independently of IL-23 (ref. 8); however, this cytokine is critical for their subsequent survival, expansion and consequent acquisition of pathogenicity^6^,^7^,^8^. Thus, we first examined the role of IL-23, in relation to TGFβ1 and IL-6, in regulation of CCR2^+^ Th17 cell development by stimulating splenocytes from d5 EAE mice *ex vivo* with MOG_35–55_ in the presence or absence of these cytokines. *Ex vivo* stimulation with MOG_35–55_ promoted generation of Th17 cells displaying a CCR6^+^CCR2^+^ phenotype, whereas addition of IL-23, and not TGFβ1/IL-6, drove development of CCR6^−^CCR2^+^ Th17 cells (Fig. 5a and Supplementary Fig. 4). To interrogate the role of IL-23 in CCR2^+^ Th17 cell development *in vivo*, we assessed mice deficient in IL-23 (B6.*Il23p19*^−/−^) or its receptor (B6.*Il23r*^gfp/gfp^). As expected^8^,^9^,^33^, Th17 cell frequency was reduced in B6.*Il23p19*^−/−^and B6.*Il23r*^gfp/gfp^ spleen (Fig. 5b,d). Notably, this reduction could essentially be accounted for by the absence of Th17 cells bearing the CCR6^−^CCR2^+^ phenotype (Fig. 5c,e). Similar results were obtained using *Il12p40*-deficient mice (Supplementary Fig. 5a,b). These processes were independent of IL-12, as B6.*Il12p35*^−/−^ mice retained WT frequencies of CCR6^−^CCR2^+^ Th17 cells (Supplementary Fig. 5c,d). To determine whether these effects of IL-23 were intrinsic to CD4^+^ T cells, we generated B6.*Il23r*^gfp/gfp^ mixed BM chimeric mice. In these mice, *Il23r*-deficient CD4^+^ T cells with a Th17 phenotype were profoundly reduced (Fig. 5f), of which those bearing a CCR6^−^CCR2^+^ profile were selectively curtailed (Fig. 5g). Furthermore, in agreement with previous reports that GM-CSF expression in T cells relies on IL-23/IL-23R^6^,^7^, *Il23r* deficiency ablated GM-CSF production by CCR6^−^CCR2^+^ Th17 cells, which was not compensated for in CCR6-expressing Th17 cell populations (Fig. 5h). Thus, our data demonstrate that CCR6^−^CCR2^+^ Th17 cell development is reliant on IL-23 and encompass GM-CSF/IFNγ-secreting Th17 cells.

Taking advantage of our novel strategy to map IL-23-driven, GM-CSF/IFNγ-producing Th17 cells *in vivo*, we next assessed the importance of key cytokines reported to shape both Th17 cell development and pathogenicity in EAE. A dual role for IL-1 in Th17 cell biology has been described: functioning as a polarizing factor for initial Th17 cell differentiation^34^ and acting on Th17 cells to promote their inflammatory potential^6^. Accordingly, neutralization of IL-1R1 inhibited Th17 cell generation (Supplementary Fig. 6a) and shifted the balance towards the CCR6^+^CCR2^+^ phenotype and away from the CCR6^−^CCR2^+^ phenotype (Supplementary Fig. 6b).

TNFα plays little to no role in Th17 lineage commitment^35^, but is reported to promote *in vitro* generation of GM-CSF^+^ Th17 cells^6^. *Tnf* deficiency reduced Th17 cell development (Supplementary Fig. 7a), with a modest defect in CCR6^−^CCR2^+^ Th17 cell frequency (Supplementary Fig. 7b). Mixed BM chimera experiments revealed that this reduction in Th17 cell development was not due to T-cell intrinsic TNF receptor (TNFR)1 or TNFR2 function (Supplementary Fig. 7c,e); however, CCR6^–^CCR2^+^ Th17 cell development required T-cell intrinsic TNFR1, but not TNFR2 signalling (Supplementary Fig. 6d,f). Further, TNFR1 signalling was shown to promote CCR6^−^CCR2^+^ Th17 expression of GM-CSF, although conversely TNFR1 or TNFR2 signalling inhibited IFNγ expression (Supplementary Fig. 7g,h).

IFNγ is reported to inhibit Th17 cell differentiation from naive precursors^36^,^37^, but has also been shown to promote development of IFNγ^+^T-bet^+^ Th17 cells from committed Th17 cells^38^. *Ifngr* deficiency enhanced Th17 cell differentiation (Supplementary Fig. 8a), with a specific increase in generation of CCR6^−^CCR2^+^ Th17 cells (Supplementary Fig. 8b). Similar results were obtained using neutralizing antibodies to IFNγ (Supplementary Fig. 8c), suggesting that IFNγ selectively suppresses CCR6^−^CCR2^+^ Th17 cell generation *in vivo*. However, assessment of *Ifngr*-deficient Th17 cells in mixed BM chimeras revealed that IFNγ promotes the development of CCR6^−^CCR2^+^ Th17 cells in a T-cell intrinsic manner (Supplementary Fig. 8d,e). In line with this, IFNγ expression in CCR6^−^CCR2^+^ Th17 cells was also promoted by T-cell intrinsic IFNγ/IFNγR signalling (Supplementary Fig. 8f).

Together, these experiments demonstrate that IL-23 drives the later emergence of the CCR6^−^CCR2^+^ Th17 cell population, that the CCR6^−^CCR2^+^ signature defines IL-23-driven GM-CSF/IFNγ-producing Th17 cell development, that IL-1 and TNFα play important accessory roles in CCR6^−^CCR2^+^ Th17 cell differentiation, and that IFNγ plays a dual role in Th17 biology, acting on non-CD4^+^ T cells to indirectly inhibit CCR6^−^CCR2^+^ Th17 differentiation, while also directly promoting their development in a T-cell intrinsic manner.

### T-bet and Eomes drive CCR6^−^CCR2^+^ Th17 cell formation

**Figure 6:**
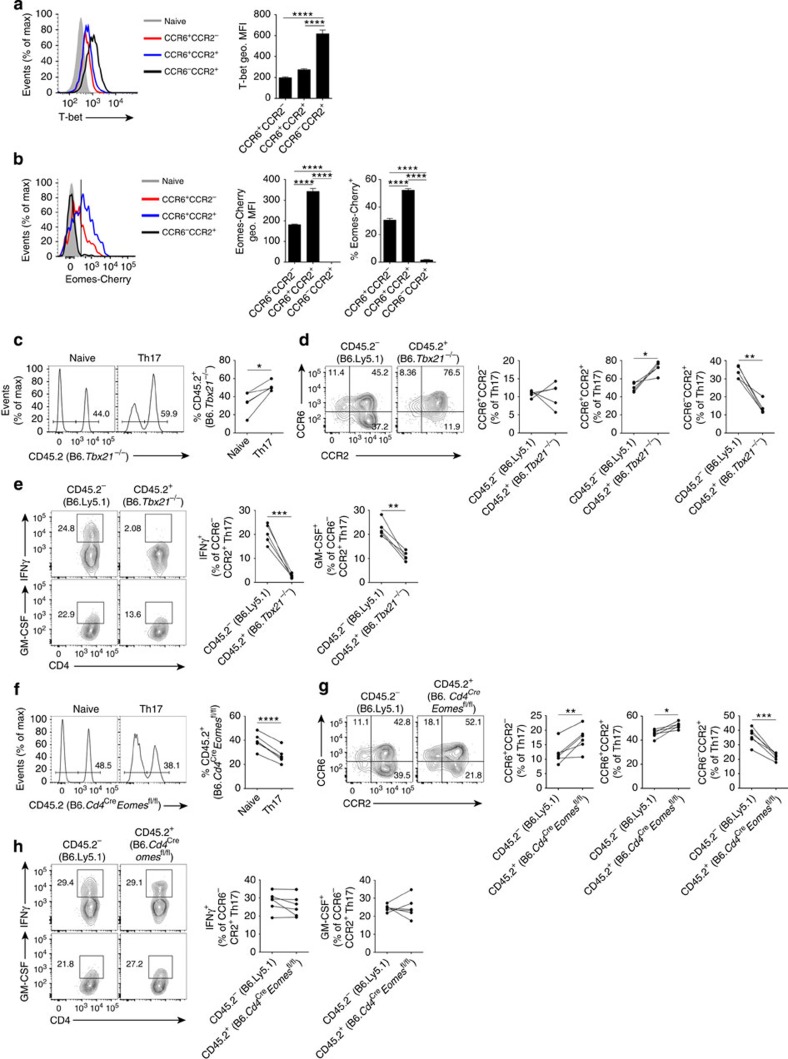
T-bet and Eomesodermin promote CCR6^−^CCR2^+^ Th17 cell differentiation *in vivo*. (**a**) Representative flow cytometric analysis and quantification (geometric MFI) of T-bet expression in naive CD4^+^ T cells (grey, filled; CD3^+^CD4^+^CD44^lo^) and CCR6^+^CCR2^−^ (red, open), CCR6^+^CCR2^+^ (blue, open) and CCR6^−^CCR2^+^ (black, open) Th17 cells (CD3^+^CD4^+^CD44^hi^IL-17A^+^) from the spleen d10 post MOG/CFA immunization. Geometric MFI (gMFI) of T-bet expression in Th17 cell populations is presented after subtraction from concurrent naive CD4^+^ T-cell T-bet gMFI. Data are representative of two independent experiments with *n*=4–5 mice per experiment. (**b**) Representative flow cytometric analysis of Eomes-Cherry expression in naive CD4^+^ T cells, CCR6^+^CCR2^−^, CCR6^+^CCR2^+^ and CCR6^−^CCR2^+^ Th17 cells (all gated and presented as in **a**) from the spleen of B6.*Eomes*^Cherry/+^ reporter mice d10 post MOG/CFA immunization. Data are representative of two independent experiments with *n*=4 mice per experiment. (**c**,**f**) Representative flow cytometric analysis and quantification of CD45.2^+^ cells within naive CD4^+^ (CD3^+^CD4^+^CD44^lo^) and Th17 cells (CD3^+^CD4^+^CD44^hi^IL-17A^+^) in the spleen of B6*.Tbx21*^−/−^ (*n*=5) (**c**) and B6.*Cd4*^Cre^*Eomes*^fl/fl^ (*n*=6) (**f**) mixed BM chimeric mice immunized with MOG/CFA 10 days prior. Data are representative of two independent experiments. (**d**,**g**) Representative flow cytometric analysis and quantification of CCR6/CCR2 staining on CD45.2^−^ (B6.Ly5.1) and CD45.2^+^ (indicated KO) Th17 cells in B6.*Tbx21*^−/−^ (**d**) and B6.*Cd4*^Cre^*Eomes*^fl/fl^ (**g**) mixed BM chimeras d10 post MOG/CFA immunization. Data are representative of two independent experiments. (**e**,**h**) Representative flow cytometric analysis and quantification of IFNγ and GM-CSF staining within CD45.2^−^ (B6.Ly5.1) and CD45.2^+^ (indicated KO) CCR6^−^CCR2^+^ Th17 cells (CD3^+^CD4^+^CD44^hi^IL-17A^+^) in B6.*Tbx21*^−/−^ (**e**) and B6.*Cd4*^Cre^*Eomes*^fl/fl^ (**h**) mixed BM chimeras d10 post MOG/CFA immunization. Data are representative of two independent experiments. (**b**–**h**) *P≤0.05, **P≤0.01, ***P≤0.001, ****P≤0.0001. (**b**,**c**) Data are presented as mean±s.e.m.; one-way analysis of variance with Bonferroni multiple comparisons test. (**d**–**h**) Each dot represents an individual mouse; paired two-tailed Student's *t*-test.

To provide new insights into the transcriptional regulation of these distinct Th17 cell phenotypes *in vivo*, we screened for the expression of key transcription factors reported to direct Th17 cell differentiation in CCR6/CCR2-expressing Th17 types. A defining feature of *in vitro*-generated pathogenic Th17 cells is expression of T-bet^3^,^27^, whereas the transcriptional activators of *Il10*, c-Maf and AHR are abundant in TGFβ1/IL-6-induced *in vitro*-generated IL-10-producing Th17 cells^3^,^4^,^39^. Accordingly, high expression of T-bet was apparent in CCR6^−^CCR2^+^ Th17 cells (Fig. 6a), whereas c-Maf and Aryl hydrocarbon receptor (AHR) were abundant in the CCR6^+^CCR2^+^ Th17 population (Supplementary Fig. 9). Moreover, the expression of IRF4 and BATF, essential mediators of early specification of Th17 cells from naive precursors^40^, was highest in CCR6^+^CCR2^+^ Th17 cells (Supplementary Fig. 9). RORγt expression was marginally lower in CCR6^−^CCR2^+^ Th17 cells than other Th17 populations (Supplementary Fig. 8). Notably, novel *Eomes*-Cherry reporter mice (Supplementary Fig. 10) revealed that, among Th17 cells, the expression of Eomesodermin was confined to CCR6-expressing Th17 cell populations (Fig. 6b).

Given that T-bet and Eomesodermin were differentially expressed in CCR6^+^ and CCR6^−^CCR2^+^ Th17 cells, we examined the T-cell intrinsic function of these transcription factors using mixed BM chimeras. T-bet negatively regulates Th17 cell development from naive precursors^41^ but its function in pathogenic Th17 cell biology is contentious^27^,^42^. Consistent with prior reports^27^,^41^, we found that *Tbx21* deficiency increased Th17 cell frequency in a T-cell intrinsic manner (Fig. 6c). Assessment of CCR6/CCR2 expression on *Tbx21*-deficient Th17 cells revealed that T-bet is critical for the development of CCR6^−^CCR2^+^ Th17 cells (Fig. 6d). Moreover, *Tbx21* deficiency ablated IFNγ production and reduced GM-CSF expression by CCR6^−^CCR2^+^ Th17 cells (Fig. 6e), implicating T-bet as a crucial regulator of CCR6^−^CCR2^+^ GM-CSF/IFNγ-producing Th17 cell development *in vivo*.

The function of Eomesodermin in Th17 cell differentiation *in vivo*, to our knowledge, is unknown. *Eomes* deficiency in CD4^+^ T cells using B6.*Cd4*^Cre^*Eomes*^fl/fl^-mixed BM chimeras reduced Th17 cell generation in a T-cell intrinsic manner (Fig. 6f), identifying Eomesodermin as a novel regulator of Th17 cell development *in vivo*. Strikingly, despite abundant expression in CCR6-expressing Th17 cells, deletion of *Eomes* curtailed the development of CCR6^−^CCR2^+^ Th17 cells (Fig. 6g), but did not alter IFNγ or GM-CSF production by these cells (Fig. 6h). Collectively, these data indicate that T-bet negatively regulates differentiation of IL-17-secreting CD4^+^ T cells, but is required for the ontogeny of GM-CSF/IFNγ-producing CCR6^−^CCR2^+^ Th17 cells, whereas Eomesodermin is required for Th17 cell differentiation *in vivo* by promoting CCR6^−^CCR2^+^ Th17 cell development.

## Discussion

In the present study, we demonstrate that CCR2 is a critical driver of encephalitogenic GM-CSF-producing Th17 cell recruitment to the CNS in EAE. Further, we show that CCR6 functions to promote homing of Th17 cells only during initial phases of inflammation and is more critically required for Treg trafficking. This ‘switch' from CCR6 to CCR2 usage by Th17 cells appeared to be temporally regulated during priming as the earliest Th17 cells predominantly expressed CCR6, followed by later emergence of CCR6^+^CCR2^+^ and CCR6^−^CCR2^+^ populations in SLOs. The latter population required IL-23 and, to a lesser extent, IL-1, TNFα and IFNγ, and the transcriptional regulators T-bet and Eomesodermin for development. Assessment of cytokine expression among Th17 populations in humans and in murine models of autoimmunity and persistent bacterial infection revealed that CCR6^−^CCR2^+^ Th17 cells align with previously described GM-CSF^+^/IFNγ^+^ pathogenic Th17 cells, while CCR6^+^CCR2^+^ Th17 cells resemble previously reported Th17 cells with a more limited pathogenic potential. Thus, we define a molecular mechanism governing encephalitogenic Th17 cell recruitment to the CNS and identify unique cell surface signatures and differentiation requirements of phenotypically distinct Th17 cells *in vivo.*

Manipulation of the chemokine system has been considered a tractable target for therapeutic intervention in CD4^+^ T-cell-driven immunopathologies for many years^43^. Central to the rational design of such approaches is a detailed understanding of unique spatio-temporal homing signals used by inflammatory and regulatory subsets of T cells to infiltrate lesions. Although CCR2 has been reported to be expressed on subsets of T cells previously^44^,^45^, until now the functional significance of this was unknown. With regard to Th17 migration, most focus has fallen on CCR6 with an early report demonstrating a critical requirement for this receptor in encephalitogenic T-cell migration in EAE^18^. However, this has been challenged with the results of more recent studies demonstrating a largely redundant role for CCR6 in EAE pathogenesis^19^,^20^. Our data demonstrate that CCR6 promotes early infiltration of Th17 cells, but this is dispensable for the development of EAE, which is driven by CCR2-dependent recruitment of encephalitogenic Th17 cells. However, our experiments using mice with *Ccr6*^−/−^*Ccr2*^−/−^ T cells indicate that when T cells lack CCR6, pathological inflammation ensues even in the absence of CCR2 on T cells. This indicates that although CCR2 strongly promotes encephalitogenic T-cell recruitment to the CNS, a degree of CCR2-independent recruitment of encephalitogenic T cells must also occur, but these cells are constrained from causing disease in a CCR6-dependent manner, probably by CCR6^+^ Tregs. From a clinical perspective, our data, and those of others^19^,^20^, suggest that therapeutic targeting of CCR6 will have detrimental effects on Treg function without restraining pathogenic T cells and emphasize CCR2 as a prospective target for the treatment of inflammatory T-cell-driven pathologies such as MS. This notion is strengthened by our findings that GM-CSF- and IFNγ-producing Th17 cells bear a CCR6^−^CCR2^+^ phenotype in humans, other studies demonstrating that IFNγ-producing Th17 cells are preferentially recruited in MS lesions^31^, the observation that IL-17A/GM-CSF co-expressing CD4^+^ T cells are enriched in MS brain lesions^32^ and the well-established dependency on CCR2 for monocyte infiltration of the CNS^46^.

Our data indicate that initial Th17 cells differentiate in an IL-23-independent manner, bear a CCR6^+^CCR2^−^ phenotype and are recruited to the uninflamed CNS via CCR6. Reboldi *et al.*^18^ proposed that an early CCR6-dependent wave of Th17 cells initiates CNS inflammation in EAE. CCR6^+^CCR2^−^ Th17 cells express a unique cytokine profile including IL-17A/F, IL-22, IL-2, TNFα and IL-10, and although mice deficient in *Il17a*, *Il17f*, *Il22* or *Tnf* do not display substantial defects in EAE pathogenesis^47^,^48^,^49^, it is possible that these CCR6^+^CCR2^−^ Th17 cell-derived factors may synergistically contribute to the initiation of CNS inflammation. Importantly however, our data clearly demonstrate that the absence of this CCR6-driven wave of Th17 cells does not prevent subsequent CCR2-driven population of the CNS by encephalitogenic Th17 cells or the development of clinical EAE, challenging notions that these cells form an essential component of EAE pathogenesis.

Subsequent to the generation of CCR6^+^CCR2^−^ Th17 cells is the emergence of CCR2-expressing Th17 cell populations. CCR6^+^CCR2^+^ Th17 cells express IL-10 and IL-9, consistent with published descriptions of Th17 cells with a more limited pathogenic potential^2^,^3^,^4^,^5^. Conversely, CCR6^−^CCR2^+^ Th17 cells express abundant GM-CSF and IFNγ, and probably constitute the previously described pathogenic Th17 cell^3^,^4^,^6^,^7^,^11^. Th17 cells with pathogenic function are reported to derive from committed TGFβ1/IL-6-driven Th17 precursors in the presence of IL-23 (refs 6, 7, 8) and from naive precursors via TGFβ3 and IL-6 (ref. 4), or independently of TGFβ1 in an IL-6-, IL-1β- and IL-23-dependent manner^3^. Here we demonstrate that the absence of the IL-23/IL-23R axis specifically curtails the development of GM-CSF-producing CCR6^−^CCR2^+^ Th17 cells, suggesting that the CCR6^−^CCR2^+^ signature defines the IL-23-driven pathogenic/inflammatory Th17 cell subset *in vivo* that may represent an advanced differentiated state of Th17 cells that arise from early CCR6^+^ precursors. Recent reports have demonstrated that the fate of Th17 cells in chronic inflammatory settings includes transdifferentiation to an IL-17A^−^ IFNγ^+^ Th1-like phenotype (termed Th1^ex-Th17^ cells) via IL-23 (ref. 11) or an IL-10-secreting, anti-inflammatory T regulatory type-1 cell (termed Tr1^ex-Th17^ cells) via TGFβ1 (ref. 50). Our data suggest that this phenotypic segregation of Th17 cells may arise before transdifferentiation, although the relationship between IL-10-producing CCR6^+^CCR2^+^ Th17 cells, IL-23-driven GM-CSF/IFNγ-producing CCR6^−^CCR2^+^ Th17 cells and Tr1^ex-Th17^/Th1^ex-Th17^ cells remains to be determined.

Our data indicate that the transcriptional regulators T-bet and Eomesodermin drive CCR6^−^CCR2^+^ Th17 cell development *in vivo*. TGFβ1-mediated repression of Eomesodermin is required for *in vitro* Th17 differentiation^51^; however, although *Eomes* can be induced in committed TGFβ1/IL-6-driven Th17 cells by inflammatory cytokines^27^, ectopic *Eomes* expression did not promote IL-12-driven IFNγ^+^ Th17 cell development *in vitro*^27^. We found that among Th17 cells, Eomesodermin expression is restricted to CCR6-expressing Th17 cell populations but is not required for their development *in vivo*. Instead, *Eomes* deficiency led to a selective defect in CCR6^−^CCR2^+^ Th17 cell generation, implicating Eomesodermin as a key regulator of the switch from CCR6 to CCR2 expression during Th17 cell development. T-bet function in pathogenic Th17 cell biology is controversial with data indicating that these cells develop independently of T-bet^42^ and reports demonstrating that IL-23/IL-12 induce T-bet^11^,^27^, which, in collaboration with Runx1, promote conversion of Th17 precursors into pathogenic IFNγ-producing Th17 cells^27^. Our data demonstrate that in the absence of T-bet, Th17 cell development is amplified but arrested at an early developmental stage with a selective defect in CCR6^−^CCR2^+^ GM-CSF/IFNγ-producing Th17 cell formation. T-bet interactions with Runx1 suppress Runx1-mediated transactivation of *Rorc* and sequester ‘available' Runx1 that would otherwise form transcriptionally active Runx1:RORγt complexes required for *Il17a* and *Il17f* induction in CD4^+^ T cells^41^. Further, Eomesodermin directly represses *Rorc* and *Il17a* transcription^51^. Thus, we speculate that Eomesodermin and T-bet shape Th17 differentiation and plasticity by implementing changes to the transcriptional landscape of Th17 cells, such as repression of *Rorc* and *Il17a*, and induction of *Ifng* and *Csf2* (directly or indirectly), as they differentiate from IL-17A^+^ CCR6^+^CCR2^+/−^ Th17 cells, through IL-17A^+^ CCR6^−^CCR2^+^ GM-CSF/IFNγ-producing Th17 cells and perhaps towards an ‘ex-Th17' phenotype in chronic inflammation^11^.

**Figure 7:**
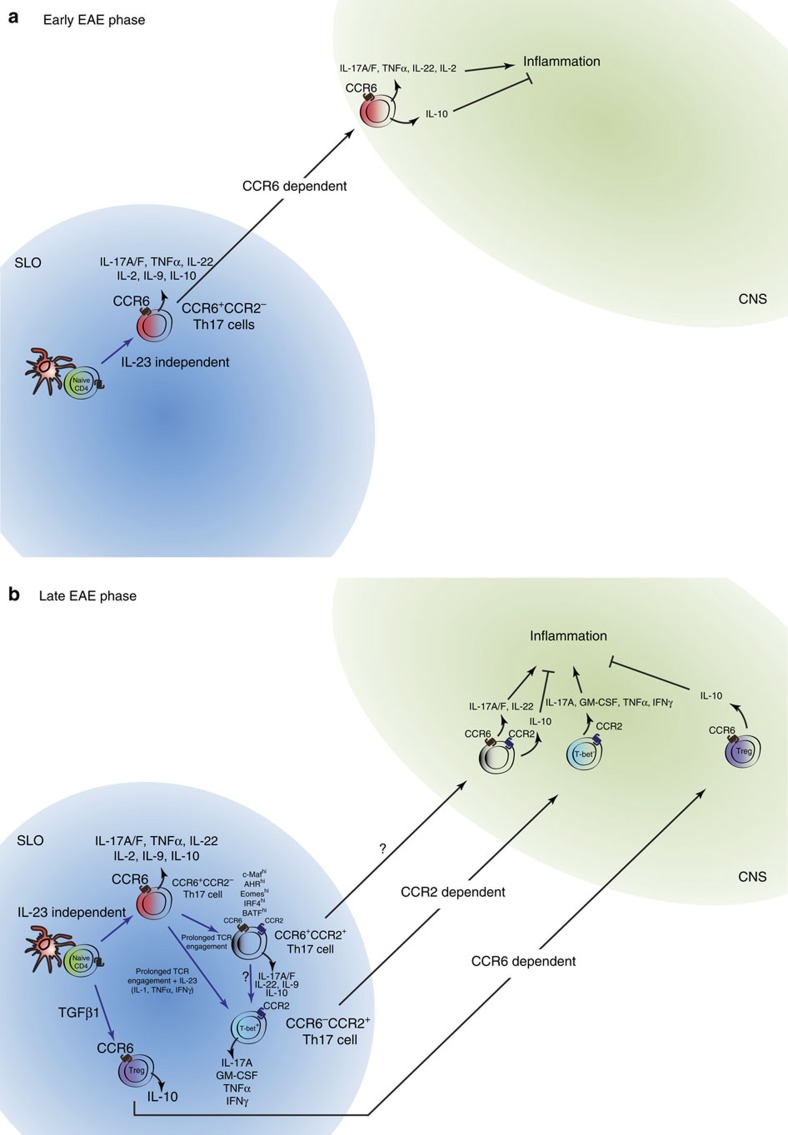
Proposed model of Th17 cell development and homing in EAE. (**a**) Initial Th17 cell differentiation from naive precursors occurs independently of IL-23 and gives rise to CCR6^+^CCR2^−^ Th17 cells. These cells gain access to the CNS via CCR6 (first wave) and may contribute to the initiation of CNS inflammation via the provision of inflammatory cytokines including IL-17A/F, TNFα, IL-22 and IL-2. (**b**) Meanwhile in the SLOs, persistent antigen drives the emergence of CCR2^+^ Th17 cell population whereby prolonged TCR stimulation gives rise to CCR6^+^CCR2^+^ Th17 cells, which express IL-10 and IL-9, whereas the presence of IL-23 and, to a lesser extent, IL-1, TNFα and IFNγ drives differentiation of GM-CSF/IFNγ-producing CCR6^−^CCR2^+^Th17 cells in a T-bet and Eomesodermin-dependent manner. Encephalitogenic Th17 cells gain access to the CNS via CCR2, independently of CCR6 (second wave), where the balance between CCR6^+^CCR2^+^ IL-10-producing Th17 cells, CCR6^−^CCR2^+^ GM-CSF/IFNγ-producing Th17 cells, other effector T cells (not depicted) and CCR6^+^ Tregs determines the outcome of disease.

Taken together, our data support a step-wise model of Th17 cell differentiation and homing (Fig. 7). Initial CCR6^+^CCR2^−^ Th17 cells develop independently of IL-23 and migrate to effector sites via CCR6. Continuing antigen exposure in SLOs drives transition of CCR6^+^CCR2^−^ Th17 cells to CCR2^+^ Th17 cell populations. More specifically, persistent antigen drives the development of CCR6^+^CCR2^+^ Th17 cells and the cytokines IL-23, IL-1, IFNγ and TNFα promote CCR6^−^CCR2^+^ GM-CSF/IFNγ-producing Th17 cells that develop in a T-bet- and Eomesodermin-dependent manner. CCR2 drives subsequent waves of Th17 cell recruitment to inflammatory sites where it is likely to be that a balance between CCR6^−^CCR2^+^ GM-CSF/IFNγ-producing Th17 cells, CCR6^+^CCR2^+^ IL-10-producing Th17 cells, other effector T-cell populations and CCR6^+^ Tregs dictates whether amplification or resolution of inflammation results. This switch from CCR6 to CCR2 as Th17 cells develop greater inflammatory potential identifies a novel temporally regulated recruitment mechanism that amplifies T-cell-dependent inflammation, a finding that has important implications for understanding regulation of autoimmune inflammation and protective immunity.

## Methods

### Mice

C57Bl/6 (B6), SJL/J, B6.Ly5.1 and B6.*Rag1*^−/−^ mice were purchased from the Animal Resource Center (WA, Australia) or bred and maintained in-house at the University of Adelaide Animal Facility. B6.*Il17a*^Cre^*Rosa26*^eYFP^ (ref. 11), B6.*Foxp3*^GFP^ (ref. 52), B6.*Ccr6*^−/−^ (ref. 53) and B6.*Ccr2*^−/−^ (ref. 54) mice were bred and maintained in-house. B6.*Il23p19*^−/−^ (ref. 55), B6.*Il23r*^gfp/gfp^ (ref. 33), B6.*Il12p40*^−/−^ (ref. 56), B6.*Il12p35*^−/−^ (ref. 57) and B6.*Ifngr*^−/−^ (ref. 58) mice were bred in-house at QIMR Berghofer Medical Research Institute, Herston, Australia. B6.*Tnf*^−/−^ (ref. 59), B6.*Tnfrsf1a*^−/−^ (ref. 60) and B6.*Tnfrsf1b*^−/−^ (ref. 61) mice were kindly provided by Professor Bernhard Baune (University of Adelaide, Adelaide, Australia). B6.*Tbx21*^−/−^ (ref. 62) and B6.*Cd4*^Cre^*Eomes*^fl/fl^ (ref. 63) mice were bred in-house at The Walter and Eliza Hall Institute of Medical Research, Parkville, Australia. B6.*Eomes*^Cherry^ reporter mice were generated (validation in Supplementary Fig. 10) at The Walter and Eliza Hall Institute of Medical Research, Parkville, Australia. B6.*Tcra*^−/−^ (ref. 64) mice were kindly provided by Professor Carola Vinuesa (John Curtin School of Medical Research, Canberra, Australia). B6.*Ccr6*^−/−^. *Ccr2*^−/−^ mice were generated and maintained in-house. All B6 lines were on the C57Bl/6J background. Male and female mice between the ages of 6–12 weeks were used in experiments. Mice in each experiment were age and gender matched. All experiments were conducted in accordance to the guidelines outlined by the Animal Ethics Committee at the University of Adelaide.

### Generation of B6.*Eomes* ^Cherry^ reporter mice

The *Eomes* targeting construct used the pKW11 vector consisting of a splice acceptor, stop codons in all reading frames, an IRES, mCherry complementary DNA, an SV40 polyadenylation signal and a *PGK-Neor* gene. Genomic DNA containing loxP flanked *Eomes* exons 2–3, containing the entire *Eomes* coding region was cloned in front of the pKW11 insert. Homology arms of 5,700 bp (5′) and 2,667 bp (3′) were amplified from an *Eomes*-containing bacterial artificial chromosome and cloned into the final targeting vector. The linear targeting vector was introduced into the *Eomes* locus by homologous recombination in C57Bl/6 embryonic stem (ES) cells. Neomycin-resistant clones were screened by Southern hybridization using 5′ (digested with Sph1, giving WT 12,293 kb and *Eomes*fl^mCherry^ 9,198 kb) probes. Targeted ES cell clones were injected into BALB/c blastocysts, to obtain chimeric founders. Germline transmission was achieved with two clones resulting in the generation of two independent lines. Founders for the reporter lines lacked the 5′ loxP site and were designated as *Eomes* reporter (*Eomes*^*mCherry/+*^).

### Human samples

Heparinized blood was collected following informed consent and rested for 4 h at 22 °C before peripheral blood mononuclear cell isolation using lymphocyte separation medium (MP Biomedicals). Peripheral blood mononuclear cells were frozen in 10% Dimethyl sulphoxide (Sigma) and stored at −80 °C before analysis. Properties of the study population are described in Supplementary Information (Supplementary Table 1). All experiments were conducted in accordance to the guidelines outlined by the ethics committee at the University of Leuven.

### BM chimeras

B6.Ly5.1 mice were lethally irradiated with 1,000 Rads and reconstituted with 4–5 × 10^6^ total BM cells intravenously (i.v.) of genotypes indicated in-text. A minimum of 8 weeks was allowed for reconstitution before experimentation.

### Experimental autoimmune encephalomyelitis

Mice on the B6 background were immunized subcutaneously with 100 μg MOG_35–55_ (GL Biochem) emulsified in 100 μl Complete Freund's Adjuvant (CFA) containing 85% mineral oil (Sigma), 15% mannide manooleate (Sigma) and 8.33 mg ml^−1^ *Mycobacterium tuberculosis* strain H37RA (Difco Laboratories). SJL/J mice were immunized subcutaneously with 100 μg proteolipid protein (PLP)_139–151_ emulsified in 100 μl CFA (as above with the addition of 0.5 mg ml^−1^ *Mycobacterium butyricum* (Difco Laboratories)). Mice received 300 ng pertussis toxin (List Biological Laboratories) intraperitoneally on days 0 and 2. For B6.*Rag1*^−/−^ EAE experiments, 8 × 10^6^ CD4^+^ T cells (CD4^+^ T-cell isolation kit, StemCell Technologies) from B6 or B6.*Ccr2*^−/−^ mice were transferred i.v. into B6.*Rag1*^−/−^ recipients, which were immunized for EAE the next day as described above.

Disease scores in MOG_35–55_-induced and PLP_139–151_-induced EAE were assigned as described previously^21^,^65^. Disease state in the SJL/J/PLP_139–151_ model was assigned as follows: peak acute disease: score ≥2; remission: previously score ≥2, now score ≤1 for minimum of 2 days; relapse: reached peak acute disease and remission, and is now score ≥2. In Fig. 2c, mice in remission were randomly allocated to experimental groups by two independent researchers by drawing an experimental group number (at equal odds) in a blinded manner. EAE disease scores were assigned in a blinded manner by two independent researchers. For isolation of CNS leukocytes, mice were perfused through the left cardiac ventricle with PBS, the brain and spinal cord harvested and dissociated through 70-μm nylon filters (BD), followed by 40% Percoll gradient centrifugation.

### *S. pneumoniae* nasopharyngeal colonization

*S. pneumoniae* strain EF3030 (serotype 19F) was obtained from Professor David E. Briles (University of Alabama at Birmingham, USA). The EF3030 strain was chosen as it causes long-term nasopharyngeal colonization in mice, with no detectable bacteremia^66^,^67^. For mouse challenge, EF3030 was grown in serum broth (nutrient broth containing 10% vol/vol heat-inactivated horse serum) at 37 °C in 5% CO_2_ to *A*_600_=0.17 (∼5 × 10^7^ CFU ml^−1^). Serotype 19F-specific capsule production was confirmed by the Quellung reaction. Bacteria were harvested by centrifugation at 3000*g*, washed once in PBS and resuspended in PBS to 5 × 10^8^ CFU ml^−1^. Mice were challenged with 10 μl (5 × 10^6^ CFU) of bacterial suspension by microtip instillation into both nares without anaesthesia. The challenge dose was confirmed retrospectively by serial dilution and plating of the inocula on blood agar.

### *In vivo* neutralization/antagonism

Mice were administered 100 μg CCL2_ala_ (scrambled peptide control)^22^,^23^, CCL2_9–76_ (CCR2 receptor antagonist)^23^ or CCL20_6–70_ (CCR6 receptor antagonist)^21^,^22^ intraperitoneally using the dosing regimen indicated in-text. Neutralizing antibodies to CCL2 (BioXCell; clone 2H5; 300 μg per dose), IFNγ (BioXCell; clone XMG1.2; 250 μg per dose) and IL-1R1 (BioXCell; clone JAMA-147; 250 μg per dose) were administered intraperitoneally using the dosing regimen indicated in-text.

### Flow cytometry

Antibodies used in this study are described in Supplementary Information (Supplementary Tables 2 and 3). For assessment of intracellular cytokine expression in murine cells, cells were first stimulated with phorbol 12-myristate 13-acetate (PMA; 20 ng ml^−1^) (Invitrogen), Ionomycin (1 μM) (Invitrogen) and GolgiStop (BD; as per the manufacturers' instructions) in complete IMDM for 4–5 h before surface staining. Dead cells were excluded using LIVE/DEAD fixable near-infrared dye (Molecular Probes). Cells were stained in PBS containing 0.04% sodium azide and 1% BSA (Sigma) or 2% FCS. Fc receptors were blocked before surface staining with 1 mg ml^−1^ murine γ-globulin (Rockland). For detection of CCR2, 10^6^ cells were stained with 5.5 μg ml^−1^ purified rat anti-mouse CCR2 (Clone MC21). Goat anti-rat IgG-Alexa Fluor 647 (Life Technologies) secondary antibody pre-adsorbed in 1% normal mouse serum and murine γ-globulin (0.5 mg ml^−1^; Rockland) was used to detect primary rat antibody. Following secondary antibody staining, cells were incubated in rat γ-globulin (1 mg ml^−1^; Rockland) before directly conjugated surface antibodies. Intracellular staining was performed using the BD Cytofix/Cytoperm kit (staining cytokines only) or eBioscience Foxp3/Transcription Factor Staining Buffer Set (transcription factor and cytokine staining) as per the manufacturers' instructions. For detection of transcription factors (excluding Foxp3), antibodies were pre-adsorbed in 2% normal mouse serum and 2% normal rat serum for 20 min before staining. Data were acquired on BD LSRII, BD FACSCanto, BD FACSAria or BD LSRFortessa flow cytometers. BD FACSAria was used for sorting experiments. For assessment of cytokine expression in human cells, thawed cells were stimulated for 5 h in 1 × Cell Stimulation Cocktail (plus protein transport inhibitors) (eBioscience). Stimulated cells were surface stained, then fixed and permeabilized using Cytofix/Cytoperm (BD) before staining for cytokines. Data were acquired on a BD FACSCantoII. All data were analysed using FlowJo software (Treestar).

### *Ex vivo* MOG-reactive Th17 cell culture and T-cell transfers

Splenocytes from d5 MOG/CFA-immunized mice were cultured (10^6^ cells per ml) in complete IMDM (Gibco) containing MOG_35–55_ (10 μg ml^−1^; GL Biochem), anti-IFNγ (10 μg ml^−1^; Clone XMG1.2, BioXCell) and anti-IL-4 (10 μg ml^−1^; Clone 11B11, BioXCell) with the addition of either no cytokines, TGFβ1 (2 ng ml^−1^; eBioscience) and IL-6 (20 ng ml^−1^; eBioscience), or IL-23 (10 ng ml^−1^; eBioscience). Cells were analysed after 3 days of culture. For transfer experiments, donor mice were immunized subcutaneously with 100 μg MOG_35–55_/CFA in footpads and hind flanks. Popliteal and inguinal lymph nodes were harvested 10 days post immunization and MOG-reactive Th17 cells expanded *ex vivo* in complete IMDM (Gibco) containing MOG_35–55_ (10 μg ml^−1^; GL Biochem) and IL-23 (3 ng ml^−1^; eBioscience) at a cell density of 10^6^ cells per ml for 3 days. Before transfer, numbers of MOG-reactive Th17 (TCRβ^+^CD4^+^IL-17A^+^) cells in culture were determined following restimulation with PMA/Ionomycin/GolgiStop in complete IMDM for 4–5 h using flow cytometry. MOG-reactive Th17 cells (3 × 10^5^) were adoptively transferred i.v. into pre-immunized congenic recipient mice as described in text.

### *Ex vivo* chemotaxis assays

Splenocytes from d10 MOG_35–55_/CFA-immunized mice were rested at 37 °C in complete RPMI 1640 for 3–4 hr. Chemokines (recombinant mouse CCL20 or recombinant mouse CCL2; kindly provided by the late Professor Ian Clark-Lewis) diluted in 150 μl chemotaxis buffer (RPMI 1640 with 0.5% BSA and 20 mM HEPES) were added to lower chambers of 96-well Transwell chemotaxis plates (5-μm pore size; Corning). Rested cells were extensively washed in chemotaxis buffer and loaded into the upper chambers at 2 × 10^6^ cells per well in 50 μl of chemotaxis buffer and incubated for 3 h at 37 °C. To enumerate Th17, Th1 and Treg cell migration, cells were harvested from the bottom chambers, restimulated in complete IMDM containing PMA/Ionomycin/GolgiStop as described above for 4 h before flow cytometric analyses. Cells of interest were gated as follows: Th1: CD3^+^CD4^+^Foxp3^−^IL-17A^−^IFNγ^+^; Th17: CD3^+^CD4^+^Foxp3^−^IL-17A^+^; Treg: CD3^+^CD4^+^IL-17A^−^IFNγ^−^Foxp3^+^. Migration index was calculated by dividing the number of positive events in test wells by the number of positive events in which no chemokine was added to the bottom chamber. Migrated cells of interest were enumerated using CaliBRITE beads (BD) as an internal reference.

### Quantitative PCR

Primer sequences used in this study are described in Supplementary Information (Supplementary Table 4). RNA was harvested from cells using the Qiagen microRNeasy kit with on-column DNase treatment as per the manufacturers' instructions. cDNA synthesis was performed using the Transcriptor First Strand cDNA synthesis kit (Roche) and used as template in reactions using LightCycler 480 SYBR Green master mix I (Roche) according to the manufacturers' instructions. Relative abundance of transcript was calculated as 2^−ΔCT^, that is, ΔCT=(CT_Target_−CT_*Rplp0*_).

### ELISA

Supernatants from homogenized CNS samples were stored at −80 °C in PBS containing a protease inhibitor cocktail (Sigma) until the day of analysis. ELISA were conducted as previously described^65^. CCL2 and CCL20 ELISA: capture and detection antibody from R&D; IL-10 and IFNγ ELISA: capture and detection antibody from eBioscience; IL-17A ELISA: capture antibody, purified clone TC11-18H10 from BD; and detection antibody, biotinylated clone TC11-8H4 from BD were used.

### Statistics

Data were analysed with Prism 6 (GraphPad Software) using two-tailed unpaired or paired Student's *t*-tests, one-way or two-way analysis of variances with appropriate post tests as indicated in text. For all analyses, *P*≤0.05 was considered significant. Sample or experiment sizes were determined empirically for sufficient statistical power. No statistical tests were used to predetermine the size of experiments. No data points were excluded from statistical tests. Statistical analysis was performed on groups with similar variance. Limited variance was observed within sample groups.

## Additional information

**How to cite this article:** Kara, E. E. *et al.* CCR2 defines *in vivo* development and homing of IL-23-driven GM-CSF-producing Th17 cells. *Nat. Commun.* 6:8644 doi: 10.1038/ncomms9644 (2015).

## Supplementary Material

Supplementary Information Supplementary Figures 1-10, Supplementary Tables 1-4

## Figures and Tables

**Table 1:** EAE disease parameters in T-cell-specific chemokine receptor-deficient BM chimeras.

**Group**	**Incidence**	**Mean day onset (**±**s.e.m.)**	**Mean max disease (**±**s.e.m.)**	**Mean cumulative disease (**±**s.e.m.)**
B6.*Tcra*^−/−^:B6	16/16	8.56±0.74	2.02±0.10	28.14±2.00
B6.*Tcra*^−/−^:B6.*Ccr6*^−/−^	18/18	11.83±0.37	2.67±0.17	26.75±2.76
B6.*Tcra*^−/−^:B6.*Ccr2*^−/−^	15/16	10.53±0.60	1.17±0.17	14.47±2.71
B6.*Tcra*^−/−^:B6.*Ccr6*^−/−^.*Ccr2*^−/−^	17/18	14.88±0.41	2.64±0.23	23.44±1.84

BM, bone marrow; EAE, experimental autoimmune encephalomyelitis

**Table 2:** EAE disease parameters in CD4^+^ T cell reconstituted *Rag1*-deficient mice.

**Group**	**Incidence**	**Mean day onset (**±**s.e.m.)**	**Mean max disease (**±**s.e.m.)**	**Mean cumulative disease (**±**s.e.m.)**
B6→B6.*Rag1*^−/−^	5/5	15.2±0.86	2.0±0.77	13.8±5.29
B6.*Ccr2*^−/−^→B6.*Rag1*^−/−^	0/5	NA	0.0±0.00	0.0±0.00

NA, not applicable.
