# Supplementary material for: CCR2 defines in vivo development and homing of IL-23-driven GM-CSF-producing Th17 cells
Source: Nat Commun. 2015 Oct 29;6:8644. doi: 10.1038/ncomms9644 (PMC4639903; doi:10.1038/ncomms9644)
Supplement: Supplementary Information — Supplementary Figures 1-10, Supplementary Tables 1-4 [file ncomms9644-s1.pdf]

SUPPLEMENTARY FIGURES

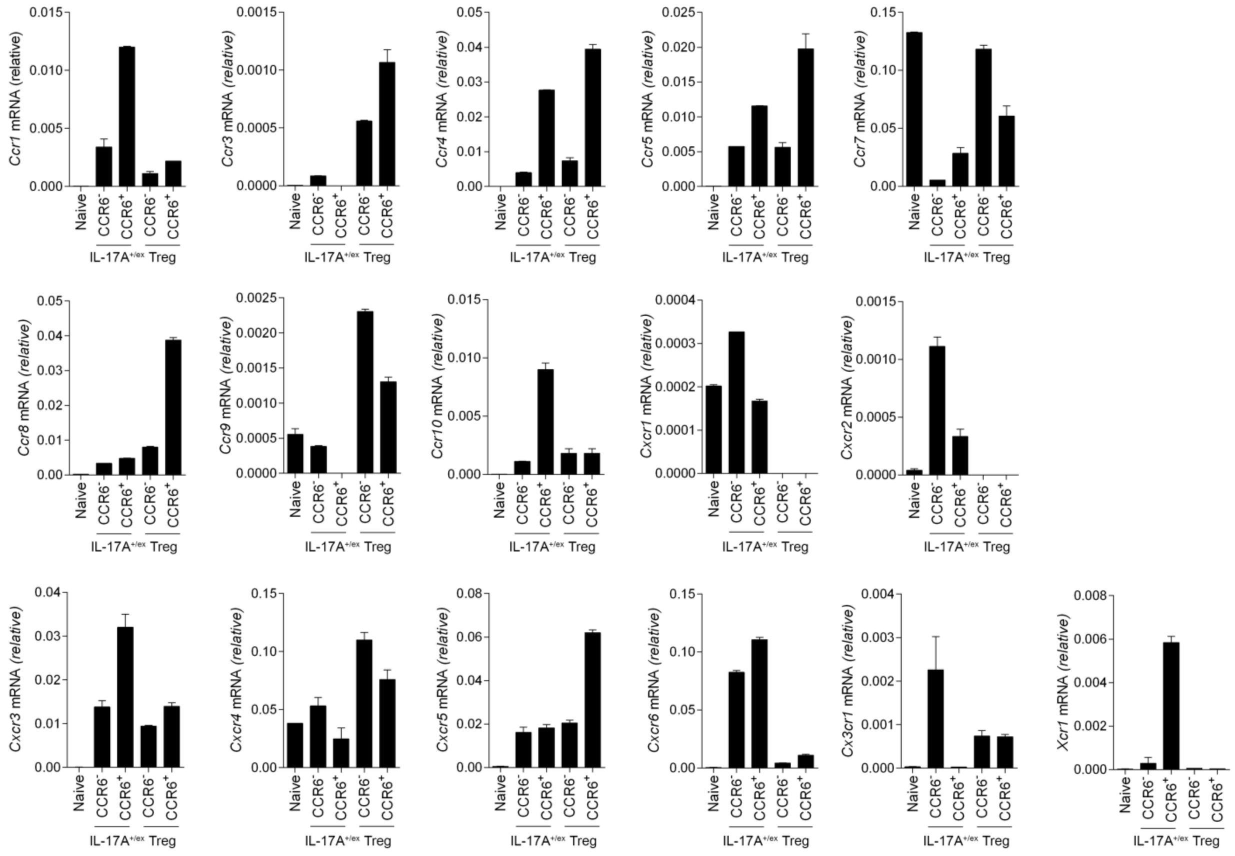

Supplementary Figure 1: Chemokine receptor expression profiles of CCR6^{+} and CCR6^{-} CD4^{+}IL-17A$^{+/ex}$ and Treg cells.

Quantitative PCR analysis of chemokine receptor transcript abundance in FACS-purified CCR6^{+} and CCR6^{-} subsets of CD4^{+}IL-17A$^{+/ex}$ cells (CD3^{+}CD4^{+}CD44^{hi}IL-17A-eYFP^{+} - B6.*Il17a*^{Cre}*Rosa26*^{eYFP} mice) and Tregs (CD3^{+}CD4^{+}Foxp3-GFP^{+} - B6.*Foxp3*^{GFP} mice) from pooled spleen and dLN of mice 10 days post MOG/CFA immunization. Data presented relative to *Rplp0* (mean ± SD).

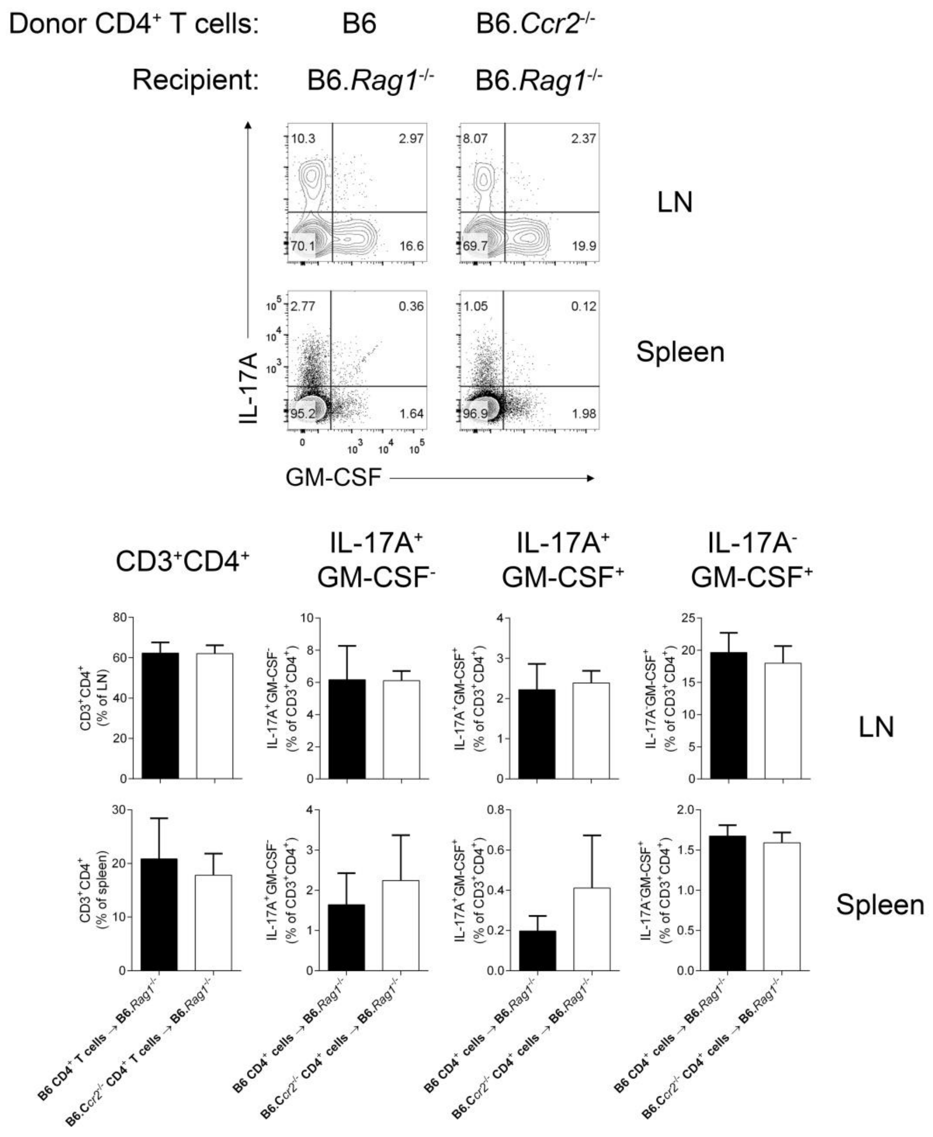

Supplementary Figure 2: *Ccr2*-deficiency on CD4$^+$ T cells does not alter Th17 cell of GM-CSF$^+$ Th cell generation in EAE-induced B6.*Rag1*$^{-/-}$ T cell-reconstituted mice.

8×10^6 MACS-purified CD4$^+$ T cells from B6 or B6.*Ccr2*$^{-/-}$ mice were transferred into B6.*Rag1*$^{-/-}$ recipients, which were immunised for EAE the next day. Representative flow cytometric analysis and quantitation of IL-17A and GM-CSF staining in CD3$^+$CD4$^+$ T cells from the lymph node (LN) and spleen 25 days post-immunisation.

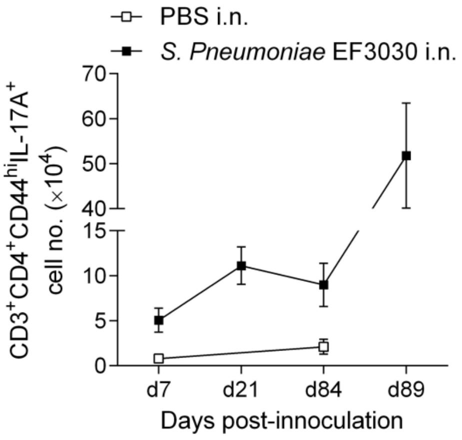

Supplementary Figure 3: Nasopharyngeal colonization with *S. pneumoniae* EF3030 induces splenic Th17 cell responses.

Mice were challenged with 5×10^6 CFU EF3030 or PBS by microtip instillation into both nares without anesthesia. Mice were sacrificed at various timepoints and Th17 cells ($CD3^+CD4^+CD44^{hi}IL\text{-}17A^+$) in the spleen enumerated over the course of infection by flow cytometry. On day 84 post-infection, mice were re-challenged i.n. with 5×10^6 CFU EF3030 and splenic Th17 cells enumerated 5 days post-secondary challenge (d89).

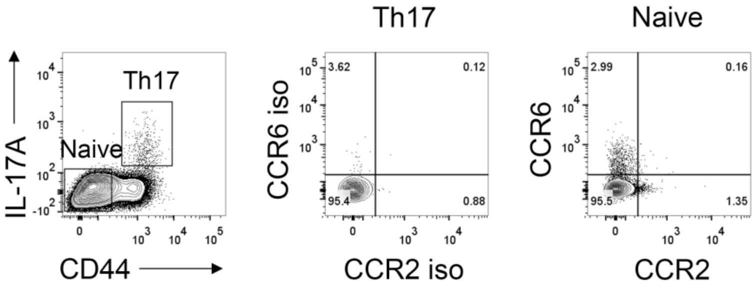

Supplementary Figure 4: Representative gating strategy relating to Fig. 5a.

Representative flow cytometric gating of naïve $CD4^+$ T cells ($CD4^+CD44^{lo}$) and Th17 cells ($CD4^+CD44^{hi}IL\text{-}17A^+$) related to Fig. 5a. Isotype staining on Th17 cells and CCR2/CCR6 expression on naïve $CD4^+$ T cells is shown.

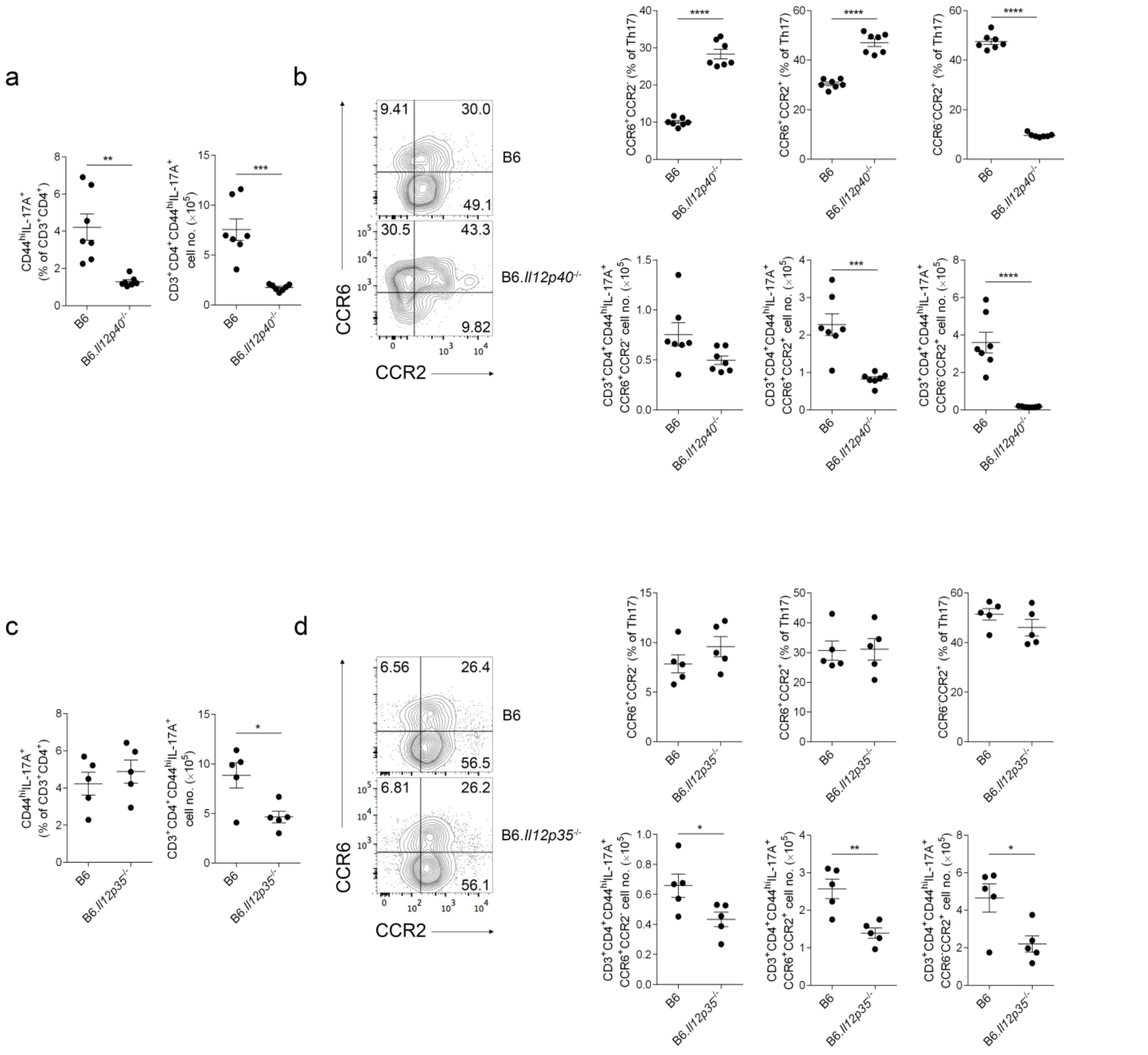

Supplementary Figure 5: Differentiation of CCR6^{-}CCR2^{+} Th17 cells *in vivo* is independent of IL-12.

(**a, b**) Analysis of B6 (n=7) and B6.*Il12p40*$^{-/-}$ (n=7) mice d10 post MOG/CFA immunization. (**a**) Frequency and total number of Th17 cells in spleen; (**b**) representative flow cytometric analysis and quantitation of CCR6 and CCR2 staining on Th17 cells. (**c, d**) Analysis of B6 (n=5) and B6.*Il12p35*$^{-/-}$ (n=5) mice d10 post MOG/CFA immunization. (**c**) Frequency and total number of

Th17 cells in spleen; (**d**) representative flow cytometric analysis and quantitation of CCR6 and CCR2 staining on Th17 cells. (**a-d**) Each dot represents an individual mouse; data presented as mean ± SEM; * $p \leq 0.05$, ** $p \leq 0.01$, *** $p \leq 0.001$, **** $p \leq 0.0001$; unpaired two-tailed Student's *t*-test.

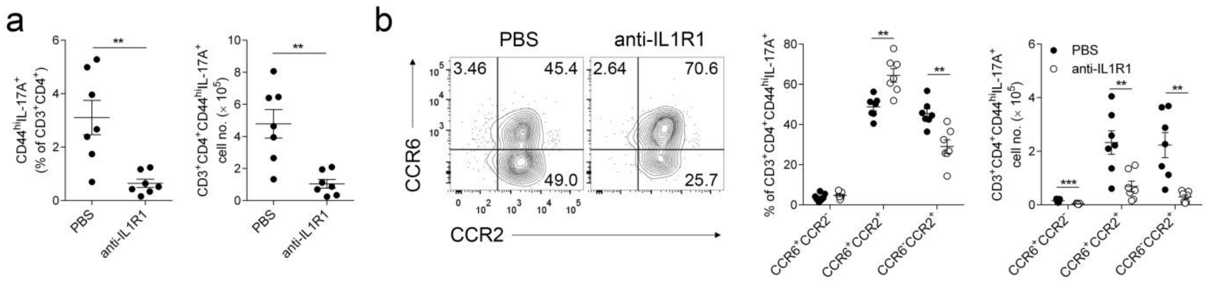

Supplementary Figure 6: IL-1 promotes CCR6$^-$CCR2$^+$ Th17 cell differentiation *in vivo*.

(**a, b**) Analysis of PBS (n=7) or neutralizing anti-IL-1R1 (n=7; 250 µg i.p. on d0 and every 48 hours thereafter) treated mice d10 post MOG/CFA immunization. (**a**) Frequency and total number of Th17 cells in spleen; (**b**) representative flow cytometric analysis and quantitation of CCR6 and CCR2 staining on Th17 cells. (**a, b**) Each dot represents an individual mouse; * p≤0.05, ** p≤0.01, *** p≤0.001; unpaired two-tailed Student's *t*-test; data presented as mean ± SEM.

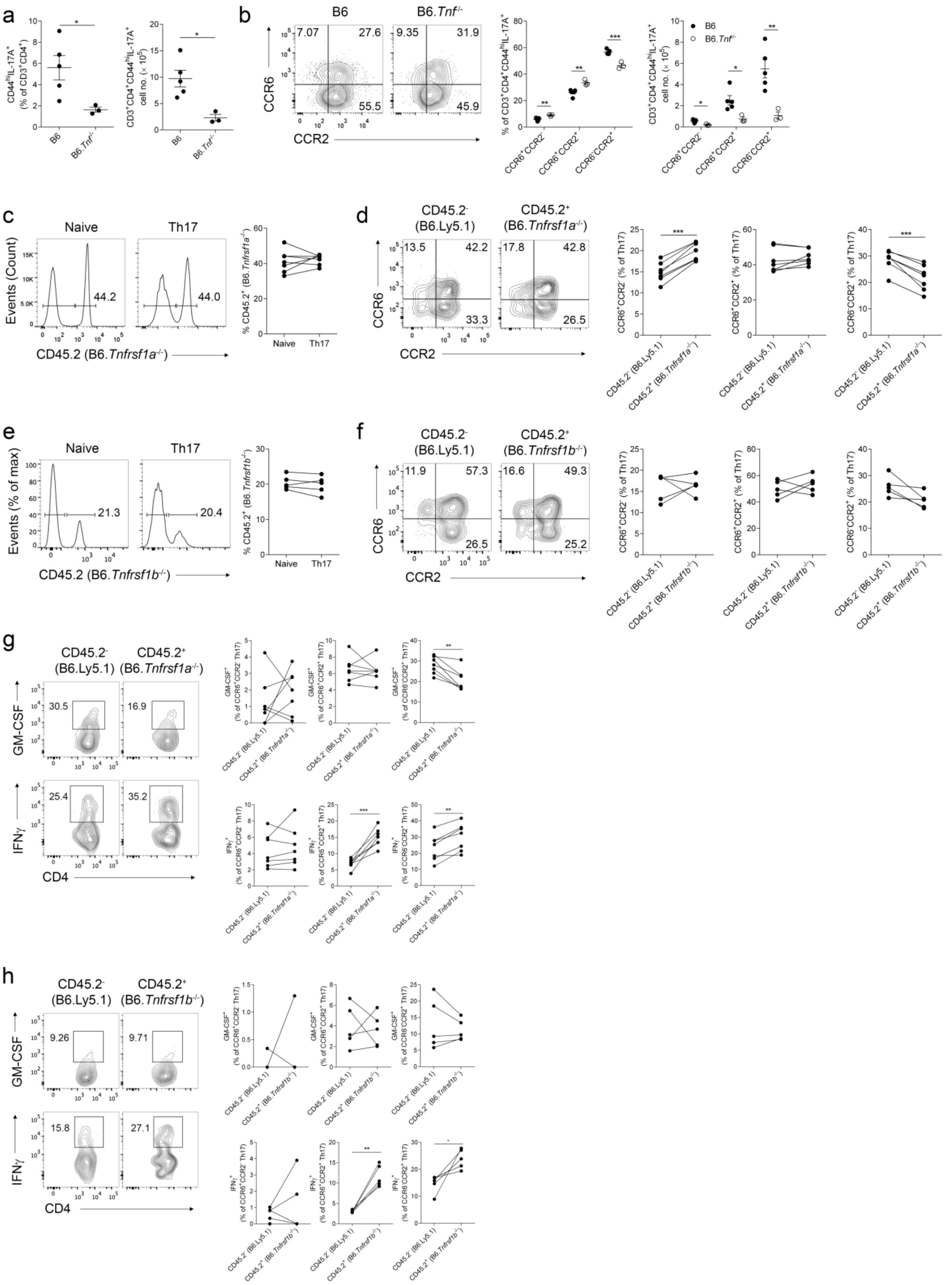
a
CD44hiIL-17A+ (% of CD3+CD4+)
CD3+CD4+CD44hiIL-17A+ cell no. (× 10^5)
B6
B6.Tnf-/-
b
B6
B6.Tnf-/-
CCR6
CCR2
7.07
27.6
55.5
9.35
31.9
45.9
% of CD3+CD4+CD44hiIL-17A+
CCR6+CCR2-
CCR6+CCR2+
CCR6-CCR2+
c
Naive
Th17
Events (Count)
44.2
44.0
CD45.2 (B6.Tnfrsf1a-/-)
% CD45.2+ (B6.Tnfrsf1a-/-)
d
CD45.2- (B6.Ly5.1)
CD45.2+ (B6.Tnfrsf1a-/-)
13.5
42.2
33.3
17.8
42.8
26.5
CCR6+CCR2- (% of Th17)
CCR6+CCR2+ (% of Th17)
CCR6-CCR2+ (% of Th17)
e
Naive
Th17
Events (% of max)
21.3
20.4
CD45.2 (B6.Tnfrsf1b-/-)
% CD45.2+ (B6.Tnfrsf1b-/-)
f
CD45.2- (B6.Ly5.1)
CD45.2+ (B6.Tnfrsf1b-/-)
11.9
57.3
26.5
16.6
49.3
25.2
g
CD45.2- (B6.Ly5.1)
CD45.2+ (B6.Tnfrsf1a-/-)
GM-CSF
30.5
16.9
IFNγ
25.4
35.2
CD4
GM-CSF+ (% of CCR6+CCR2- Th17)
GM-CSF+ (% of CCR6+CCR2+ Th17)
GM-CSF+ (% of CCR6-CCR2+ Th17)
IFNγ+ (% of CCR6+CCR2- Th17)
IFNγ+ (% of CCR6+CCR2+ Th17)
IFNγ+ (% of CCR6-CCR2+ Th17)
h
CD45.2- (B6.Ly5.1)
CD45.2+ (B6.Tnfrsf1b-/-)
GM-CSF
9.26
9.71
IFNγ
15.8
27.1
CD4

Supplementary Figure 7: T-cell expression of TNFR1 promotes $CCR6^{-}CCR2^{+}$ Th17 cell differentiation *in vivo*.

(**a ,b**) Analysis of B6 (n=5) and B6.*Tnf*$^{-/-}$ (n=3) mice d10 post MOG/CFA immunization. (**a**) Frequency and total number of Th17 cells in spleen; (**b**) representative flow cytometric analysis and quantitation of CCR6 and CCR2 staining on Th17 cells. Data are representative of 2 independent experiments. (**c, e**) Representative flow cytometric analysis and quantitation of $CD45.2^{+}$ cells within naïve $CD4^{+}$ ($CD3^{+}CD4^{+}CD44^{lo}$) and Th17 cells ($CD3^{+}CD4^{+}CD44^{hi}$IL-$17A^{+}$) in spleen of B6.*Tnfrsf1a*$^{-/-}$ (n=7) (**c**) and B6.*Tnfrsf1b*$^{-/-}$ (n=5) (**e**) mixed bone marrow chimeric mice on d10 post-MOG/CFA immunisation. (**d, f**) Representative flow cytometric analysis and quantitation of CCR6 and CCR2 staining on $CD45.2^{-}$ (B6.Ly5.1) and $CD45.2^{+}$ (**d** - B6.*Tnfrsf1a*$^{-/-}$; **f** - B6.*Tnfrsf1b*$^{-/-}$) Th17 cells in mixed bone marrow chimeras immunized with MOG/CFA 10 days prior. (**g, h**) Representative flow cytometric analysis of GM-CSF and IFNγ staining amongst $CD45.2^{-}$ (B6.Ly5.1) and $CD45.2^{+}$ (indicated KO) $CCR6^{-}CCR2^{+}$ Th17 cells in B6.*Tnfrsf1a*$^{-/-}$ (**g**, n=7) and B6.*Tnfrsf1b*$^{-/-}$ (**h**, n=5) mixed bone marrow chimeric mice 10 days post MOG/CFA immunization. Right, GM-CSF and IFNγ expression amongst CCR6/CCR2 Th17 cell populations in mixed bone marrow chimeras. (**a-h**) Each dot represents an individual mouse; * $p \leq 0.05$; ** $p \leq 0.01$; *** $p \leq 0.001$. (**a, b**) Data presented as mean ± SEM; unpaired two-tailed Student's *t*-test. (**c-h**) Paired two-tailed Student's *t*-test.

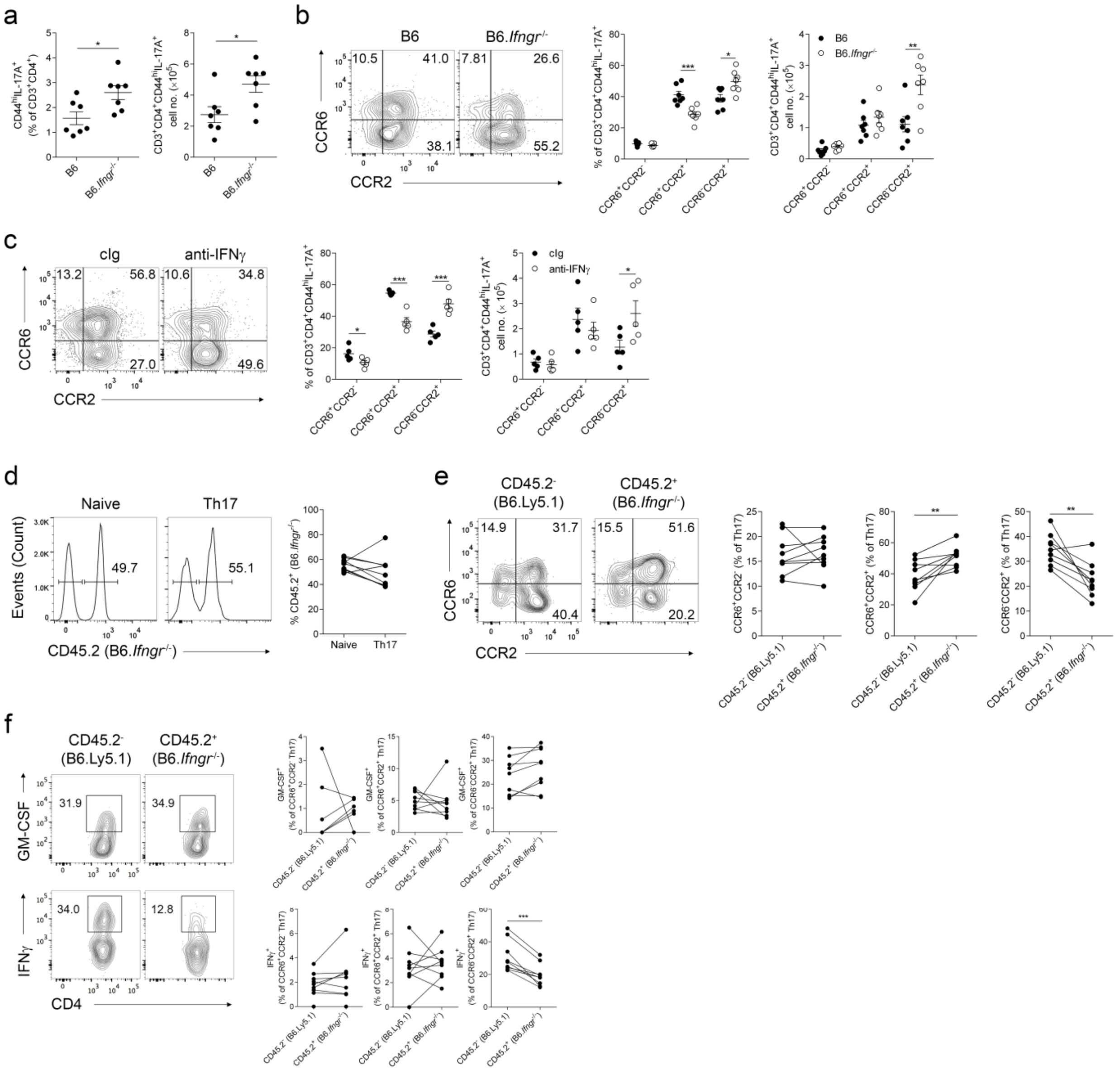

Supplementary Figure 8: T-cell intrinsic IFNγR signaling promotes CCR6^{-}CCR2^{+} Th17 cell development *in vivo*.

(**a, b**) Analysis of B6 (n=7) and B6.*Ifngr*$^{-/-}$ (n=7) mice d10 post MOG/CFA immunization. (**a**) Frequency and total number of Th17 cells in spleen; (**b**) representative flow cytometric analysis and quantitation of CCR6 and CCR2 staining on Th17 cells. (**c**) Representative flow cytometric analysis and quantitation of CCR6 and CCR2 staining on Th17 cells from control

immunoglobulin (cIg) (n=5) or neutralizing anti-IFNγ treated (n=5; 250 μg i.p. on d0 and every 48 hours thereafter) mice d10 post MOG/CFA immunization. (**d**) Representative flow cytometric analysis and quantitation of $CD45.2^{+}$ (B6.*Ifngr*$^{-/-}$) cells within naïve $CD4^{+}$ ($CD3^{+}CD4^{+}CD44^{lo}$) and Th17 cells ($CD3^{+}CD4^{+}CD44^{hi}IL\text{-}17A^{+}$) in spleen of B6.*Ifngr*$^{-/-}$ mixed bone marrow chimeric mice (n=9) immunized with MOG/CFA 10 days prior. (**e**) Representative flow cytometric analysis and quantitation of CCR6 and CCR2 staining on $CD45.2^{-}$ (B6.Ly5.1) and $CD45.2^{+}$ (B6.*Ifngr*$^{-/-}$) Th17 cells in mixed bone marrow chimeras immunized with MOG/CFA 10 days prior. (**f**) Representative flow cytometric analysis of GM-CSF and IFNγ staining amongst $CD45.2^{-}$ (B6.Ly5.1) and $CD45.2^{+}$ (B6.*Ifngr*$^{-/-}$) $CCR6^{-}CCR2^{+}$ Th17 cells in B6.*Ifngr*$^{-/-}$ (n=7) mixed bone marrow chimeric mice 10 days post MOG/CFA immunization. Right, GM-CSF and IFNγ expression amongst CCR6/CCR2 Th17 cell populations in B6.*Ifngr*$^{-/-}$ mixed bone marrow chimeras. (**a-f**) Each dot represents an individual mouse; * $p \leq 0.05$, ** $p \leq 0.01$, *** $p \leq 0.001$. (**a-c**) Data presented as mean ± SEM; unpaired two-tailed Student's *t*-test. (**d-f**) Paired two-tailed Student's *t*-test.

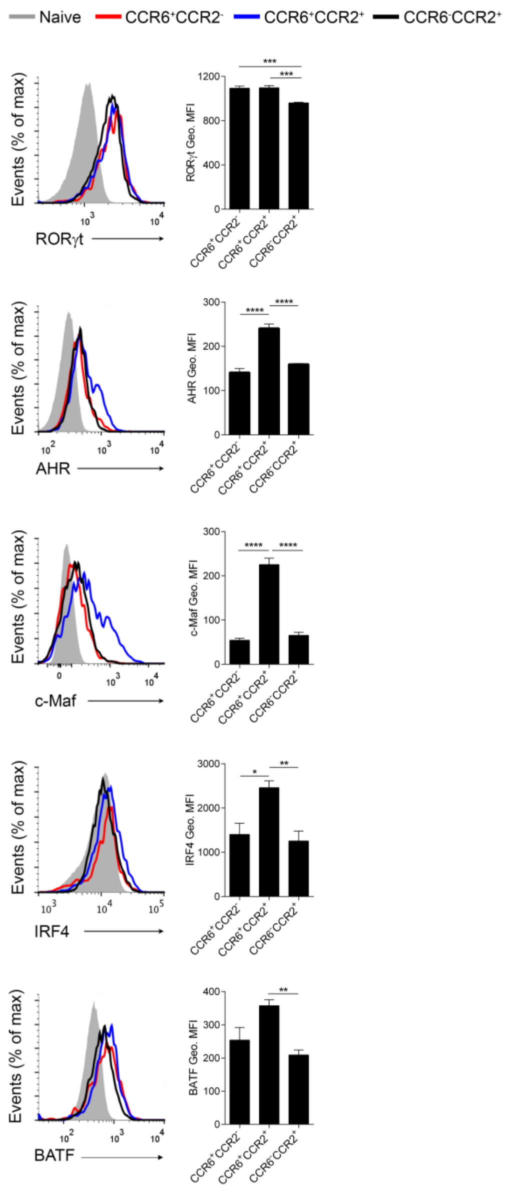

Supplementary Figure 9: Transcription factor expression by CCR6/CCR2-expressing Th17 cell populations.

Representative flow cytometric analysis and quantitation of RORγt, AHR, c-Maf, IRF4, and BATF expression in naïve $CD4^+$ T-cells (grey – filled; $CD3^+CD4^+CD44^{lo}$) and $CCR6^+CCR2^-$ (red - open), $CCR6^+CCR2^+$ (blue – open) and $CCR6^-CCR2^+$ (black – open) Th17 cells ($CD3^+CD4^+CD44^{hi}$IL-$17A^+$) from spleen d10 post MOG/CFA immunization. Geometric MFI

(gMFI) of transcription factor staining in Th17 cell populations is presented after subtraction from concurrent naïve $CD4^+$ T-cell gMFI. Data representative of 2-3 independent experiments with n=4-5 mice/experiment. * $p \leq 0.05$, ** $p \leq 0.01$, *** $p \leq 0.001$; one-way ANOVA with Bonferroni multiple comparisons test.

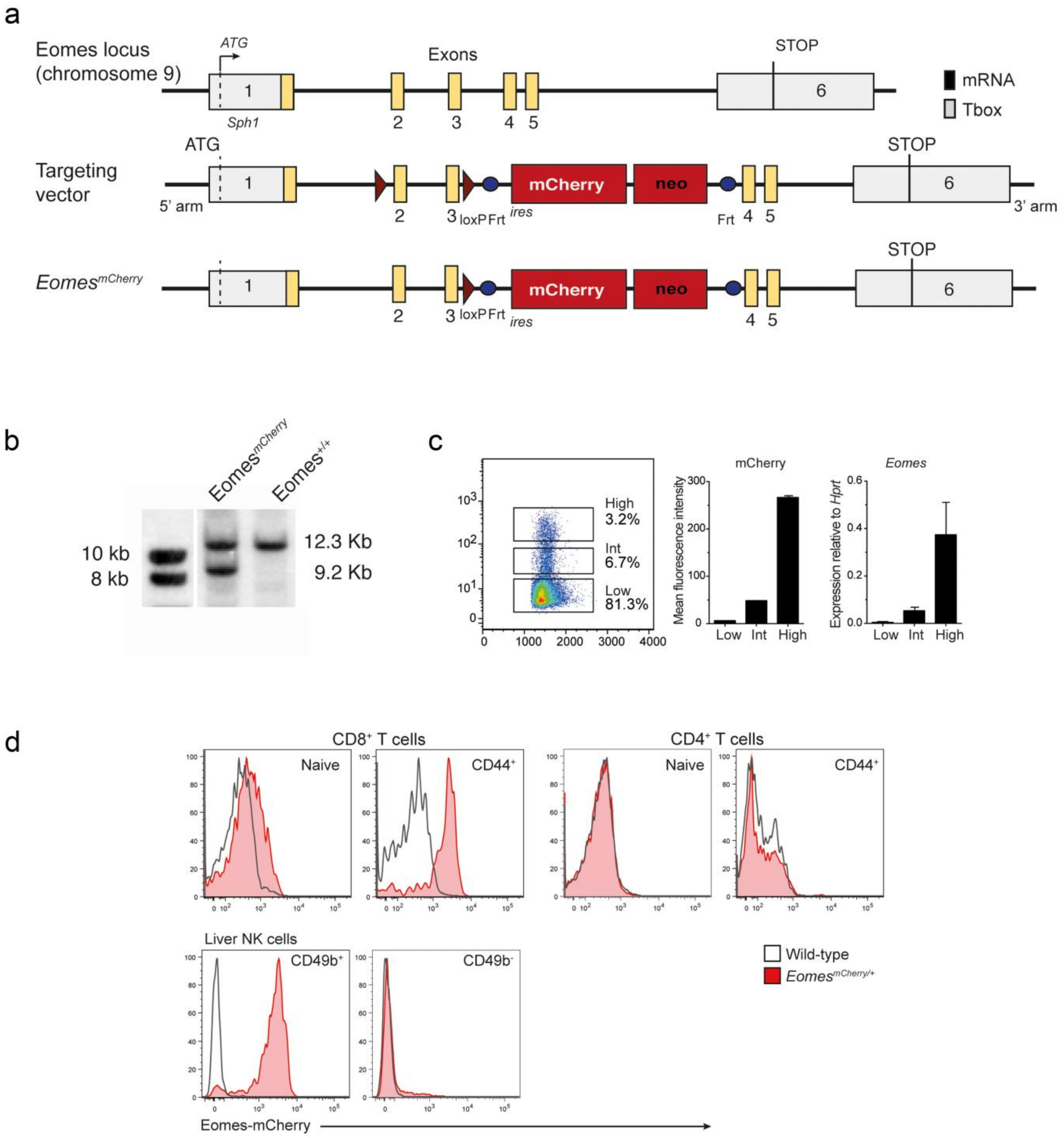

Supplementary Figure 10: B6.*Eomes*^{Cherry} reporter mice.

(**a**) The genomic locus of Eomes. Exons are represented by boxes; introns are represented as black lines; coding regions are shaded yellow; non-translated regions are in white; arrows indicate the direction of translation. The alleles derived from the integration of the targeting vector and subsequent manipulations are shown. Circles, Frt sites; triangles, loxP sites. The

Eomes-mCherry reporter line was derived from an embryonic stem cell (ES) clone that lacked the 5’ loxP site and was identified by PCR. (**b**) Southern blot analysis using a 5’ probe of ES cell Sph1-digested DNA showing the wild-type (12.293 kb) and targeted (9.198 kb) alleles. (**c**) Quantitative analysis of *Eomes* mRNA expression relative to *Hprt* for the indicated transcripts of live (PI^-) cells from splenocytes purified on the basis of their expression of Eomes-mCherry. Data show the mean ± S.D. of two experiments. (**d**) Expression of Eomes-mCherry in splenic T cell (CD8 and CD4) and hepatic NK cell subsets from wild-type (B6) and B6.$Eomes^{Cherry/+}$ mice as indicated.

SUPPLEMENTARY TABLES

Supplementary Table 1: Properties of the human study population

	**Controls**	**MS**
Total number of participants (n)	7	12
Gender (% Female)	72	75
Age at onset (years; mean (range))	-	34 (21-50)
Age at sampling (years; mean (range))	36 (30-43)	50 (27-77)
Disease duration at sampling (years; mean (range))	-	16 (6-39)
Disease course MS (% RRMS)	-	75
Duration treatment at sampling (years; mean (range))	-	Untreated

Supplementary Table 2: Anti-mouse antibodies

Anti-mouse antibodies				
**Anti-**	**Fluorophore**	**Clone**	**Source**	**Final concentration**
**CD3ε**	FITC<br>PE-Cy7<br>Biotin	145-2C11	BD<br>eBiosciences<br>eBiosciences	2.77 µg/ml<br>0.83 µg/ml<br>2.08 µg/ml
**CD4**	BV450<br>PerCP-Cy5.5<br>PE-Cy7<br>Alexa Fluor 647<br>PECF594	RM4-5	BD<br>BD<br>BD<br>BD<br>BD	0.83 µg/ml<br>0.83 µg/ml<br>0.83 µg/ml<br>0.83 µg/ml<br>0.83 µg/ml
**CD8α**	PE-Cy7	53-6.7	BD	0.83 µg/ml
**TCRβ**	PE	H57-597	eBiosciences	0.4 µg/ml
**CD44**	BV450<br>FITC<br>Biotin	IM7	BD<br>BD<br>BD	0.83 µg/ml<br>2.08 µg/ml<br>0.83 µg/ml
**CCR6**	PE<br>Purified<br>Alexa Fluor 488	140706	R&D<br>R&D<br>R&D	8 µl neat<br>4.16 µg/ml<br>9 µl neat
**CCR2**	Purified	MC21	Prof. Matthias Mack, Universität Regensburg, Germany	5.5 µg/ml
**CD11b**	PE-Cy7	M1/70	BD	0.83 µg/ml
**F4/80**	FITC	BM8	eBiosciences	2.77 µg/ml
**Gr-1**	PE	RB6-8C5	BD	0.83 µg/ml
**CD45**	APC	30-F11	BD	0.83 µg/ml

**CD45.2**	FITC<br>PerCP<br>PerCP-Cy5.5<br>Biotin	104	BD<br>Biolegend<br>eBiosciences<br>BD	2.08 µg/ml<br>2.08 µg/ml<br>0.83 µg/ml<br>2.08 µg/ml
**IL-17A**	BV510<br>PerCP-Cy5.5<br>PE	Tc11-18H10.1<br>eBio17B1<br>TC11-18H10	Biolegend<br>eBiosciences<br>BD	1.33 µg/ml<br>1.11 µg/ml<br>1.11 µg/ml
**IFNγ**	FITC<br>PE-Cy7	XMG1.2	BD<br>eBiosciences	2.77 µg/ml<br>1.11 µg/ml
**GM-CSF**	FITC	MP1-22E9	eBioscienes	2.77 µg/ml
**TNFα**	FITC	MP6-XT22	eBiosciences	2.77 µg/ml
**IL-2**	Alexa Fluor 488	JES6-5H4	eBiosciences	2.77 µg/ml
**IL-9**	PE	D9302C12	BD	1.66 µg/ml
**IL-10**	FITC	JES5-16E3	BD	4.0 µg/ml
**IL-17F**	Alexa Fluor 488	eBio18F10	eBiosciences	2.77 µg/ml
**IL-22**	PerCP-eFluor710	1H8PWSR	eBiosciences	1.66 µg/ml
**Foxp3**	PerCP-Cy5.5	FJK-16s	eBiosciences	1.66 µg/ml
**T-bet**	PerCP-Cy5.5	eBio4B10	eBiosciences	1.66 µg/ml
**RORγt**	PerCP-eFluor710	B2D	eBiosciences	1.66 µg/ml
**AHR**	Alexa Fluor 488	4MEJJ	eBiosciences	4.16 µg/ml
**c-Maf**	PerCP-eFluor710	symOF1	eBiosciences	5 µl neat
**IRF4**	PerCP-eFluor710	3E4	eBiosciences	1.66 µg/ml
**BATF**	PerCP-eFluor710	MBM7C7	eBiosciences	5 µl neat

Supplementary Table 3: Anti-human antibodies

Anti-human antibodies				
**Anti-**	**Fluorophore**	**Clone**	**Source**	**Final concentration**
**CD4**	APC-eFluor780	RPA-T4	eBioscience	8.33 μg/ml
**CD8α**	PerCP-eFluor710	SK1	eBioscience	0.20 μg/ml
**IL-17A**	FITC	eBio64DEC17	eBioscience	1.25 μg/ml
**IFNγ**	eFluor450	4S.B3	eBioscience	5.00 μg/ml
**GM-CSF**	APC	BVD2.21C11	Biolegend	5.00 μg/ml
**CCR2**	PE	K036C2	Biolegend	10.0 μg/ml
**CCR6**	PE-Cy7	G034E3	Biolegend	10.0 μg/ml

Supplementary Table 4: Primer sequences

Primer	Forward (5'-3')	Reverse (5'-3')
Rplp0	TGCAGATCGGGTACCCAACT	ACGCGCTTGTACCCATTGA
Ccr1	TGGGAGTTCACTCACCGTACCT	TCCACTGCTTCAGGCTCTTGT
Ccr2	GTTCATCCACGGCATACTATCAAC	GCCCCTTCATCAAGCTCTTG
Ccr3	TTGCCTACACCCACTGCTGTAT	TTTCCGGAACCTCTCACCAA
Ccr4	GCAACACTGCAAGAATGAGAAGA	GACCACCACGGCGAAGAT
Ccr5	CATCCGTTCCCCCTACAAGA	GGAACTGACCCTTGAAAATCCA
Ccr6	CCTGGGCAACATTATGGTGGT	CAGAACGGTAGGGTGAGGACA
Ccr7	CATTGCCTATGACGTCACCTACA	GAAGGCATACCAGAAAGGGTTGA
Ccr8	GCTCGCTCAGATAATTGGTCTTC	CGTGACGTTGGGCTCCAT
Ccr9	CAGTTCTGAGGAGGATGCTTGA	AACCCAGCTGCACTGATGATC
Ccr10	CCTCTACTCGGCCTCTTTCCA	CGGTCGGCGCTGATACAG
Cxcr1	TGTCCCACATATTTGGCTTCCT	GCCCGTAGCAGACCAGCAT
Cxcr2	GCCCTGACCTTGCCTGTCT	TGCACAGGGTTGAGCCAAA
Cxcr3	TACCTTGAGGTTAGTGAACGTCA	CGCTCTCGTTTTCCCCATAATC
Cxcr4	ACCTCTACAGCAGCGTTCTCATC	TGTTGGTGGCGTGGACAATA
Cxcr5	GGGCTCCATCACATACAATATGG	GAATCTCCGTGCTGTTACTGTAGAAG
Cxcr6	CCGGCAGGCTAAGTGGAA	CACCCAAATGAGCAAGCAAA
Cx3cr1	ATCAGCATCGACCGGTACCT	CTGCACTGTCCGGTTGTTCAT
Xcr1	CATGACCATCCACCGATACCT	GCTGCCCACACACATGATGT
